# Near-infrared laser-irradiated upconversion nanoparticles with dexamethasone precise released for alleviating lung ischemia-reperfusion injury

**DOI:** 10.3389/fbioe.2023.1176369

**Published:** 2023-05-05

**Authors:** Xiaojing He, Zhining Li, Mengling Ye, Chen Zhao, Siyi Wu, Yi Qin, Youyuan Guo, Lu Zhang, Fei Lin

**Affiliations:** ^1^ Guangxi Medical University Cancer Hospital, Nanning, China; ^2^ Guangxi Clinical Research Center for Anesthesiology, Nanning, China; ^3^ Guangxi Engineering Research Center for Tissue & Organ Injury and Repair Medicine, Nanning, China; ^4^ Guangxi Key Laboratory for Basic Science and Prevention of Perioperative Organ Disfunction, Nanning, China

**Keywords:** dexamethasone, upconversion nanoparticles, near-infrared, response-controlled release, inflammation, lung ischemia-reperfusion injury

## Abstract

**Introduction:** Dexamethasone (DEX), as an important enduring-effect glucocorticoid (GC), holds great promise in the field of lung ischemia-reperfusion injury (LIRI) comprehensive therapy owing to its immunomodulatory properties, such as inducing apoptosis and cell cycle distribution. However, its potent anti-inflammatory application is still restricted because of multiple internal physiologic barriers.

**Methods:** Herein, we developed upconversion nanoparticles (UCNPs) coated with photosensitizer/capping agent/fluorescent probe-modified mesoporous silica (UCNPs@mSiO_2_[DEX]-Py/β-CD/FITC, USDPFs) for precise DEX release synergistic LIRI comprehensive therapy. The UCNPs were designed by covering an inert YOF:Yb shell on the YOF:Yb, Tm core to achieve high-intensity blue and red upconversion emission upon Near-Infrared (NIR) laser irradiation.

**Results:** Under suitable compatibility conditions, the molecular structure of photosensitizer can be damaged along with capping agent shedding, which endowed USDPFs with an outstanding capability to carry out DEX release controlling and fluorescent indicator targeting. Furthermore, the hybrid encapsulating of DEX significantly increased utilization of nano-drugs, improving the water solubility and bioavailability, which was conducive to developing the anti-inflammatory performance of USDPFs in the complex clinical environment.

**Discussion:** The response-controlled release of DEX in the intrapulmonary microenvironment can reduce normal cell damage, which can effectively avoid the side effects of nano-drugs in anti-inflammatory application. Meanwhile, the multi-wavelength of UCNPs endowed nano-drugs with the fluorescence emission imaging capacity in an intrapulmonary microenvironment, providing precise guidance for LIRI.

## 1 Introduction

Arising after lung transplantation surgery, the pathogenesis of local acute inflammation such as lung ischemia-reperfusion injury (LIRI) is extremely complex, including an inflammatory cascade, programmed cell death, and oxidative stress(Ng et al., 2005; Ng et al., 2006). Besides, inflammatory cells (e.g., alveolar macrophages and neutrophils) are activated to secrete large amounts of inflammatory factors, which will lead to excessive uncontrolled inflammation and tissue damage(Fiser et al., 2002). Glucocorticoids (GC) are potent anti-inflammatory agents with the ability to provide a variety of anti-inflammatory, anti-edematous and pulmonary vasodilatory effects through genomic and non-genomic mechanisms(Peter et al., 2008; Coutinho and Chapman, 2011; Zhang et al., 2015). Dexamethasone (DEX), one of the most commonly used glucocorticoids, has been shown in clinical data to reduce tissue injury by decreasing the secretion of pro-inflammatory factors and inhibiting apoptosis (Aktas et al., 2017). Postoperative studies in LIRI have shown that DEX not only reduces sepsis-related lung damage (Kuwajima et al., 2019), but also reduces inflammation by attenuating the activity of the JAK2/STAT3 signaling pathway and increasing the expression of ICAM-1, thereby protecting lungs from severe acute pancreas inflammation-related lung injury (Han et al., 2016). For this reason, DEX is one of the good alternative drugs used to ameliorate the local acute inflammation generated after LIRI. However, the clinical application of DEX is due to its poor water solubility, low bioavailability and side effects on the body (Caplan et al., 2017). Therefore, it is urgent to address the issue of DEX precise release for therapeutic use in the intrapulmonary microenvironment after LIRI.

To overcome the multiple adverse effects arising from the release of drugs *in vivo* prior to treatment, various mesoporous silica (mSiO_2_)-based nano-drug delivery approaches have been widely reported (Spahn et al., 2015; Tian et al., 2015; Eskandari et al., 2019). However, the practical application of these protocols remains limited due to the lack of control over the trace images displayed *in vivo*, as well as the amount and time of drug release. Recently, upconversion nanoparticles (UCNPs) accompanied by rare earth element (Ln) doping have shown promising prospects due to their applications in bioimaging and optical therapy features (Yuan et al., 2014; Song et al., 2022). As a suitable host material for hexagonal crystalline systems, YOFs convert near-infrared light to UV-visible light by doping with Yb^3+^/Tm^3+^ ionic couples (Yi et al., 2011; Suo et al., 2016; Lee et al., 2017). Meanwhile, the surface inert layer can further reduce the defect quenching and enhance the luminescence quality (Ma et al., 2018; Lu et al., 2019). Further, the good biocompatibility of UCNPs guarantees for biomedical applications such as drug delivery and cancer therapy (Xiang et al., 2018; Yan et al., 2021). The mSiO_2_ can be effectively coated on the surface of upconversion nanoparticles (UCNPs), which is very beneficial to achieve surface functionalization of particles and optimize their properties for biomedical applications (Liu et al., 2019). The terminal methyl pyrrole of pyrene conjugated β-cyclodextrin (β-CD), as a pair of molecular capping agent couple, is often used to seal the pores of mSiO_2_ (Indrajit et al., 2010; Wang et al., 2017). In the presence of near-infrared light absorption, the ester bond adjoining the pyrene chromophore is broken and accompanied by the shedding of β-CD, resulting in the release of drugs from the mSiO_2_ pores (Liu et al., 2019). The photoswitching effect can play a significant role in the controlled release of nano-drugs and localization imaging.

In this study, YOF:Yb^3+^/Tm^3+^ UCNPs were encapsulated in mSiO_2_, then modified by γ-Oxo-1-pyrenebutyricacid (Py) and loaded with DEX, and finally capped with β-CD, which was labeled with fluorescein isothiocyanate (FITC), to construct YOF:Yb^3+^/Tm^3+^@YOF:Yb^3+^@mSiO_2_(DEX)-Py/β-CD/FITC nanohybrids (USDPFs). Under NIR irradiation, UV light from UCNPs upconversion emission breaks the ester bond adjoining the pyrene chromophore bond and promotes the detachment of nanovalve β-CD for encapsulated drug release. As a fluorescent marker, FITC can be employed to label and trace nanoparticles *in vivo* and *in vitro*. In addition, mSiO_2_ coating and β-CD coupling can also improve the biocompatibility of nanocomposites, providing security for anti-inflammatory therapy after LIRI.

## 2 Materials and methods

### 2.1 Materials

Yttrium oxide (Y_2_O_3_), Ytterbium oxide (Yb_2_O_3_), Thulium oxide (Tm_2_O_3_), Potassium fluoride (KF), Urea (CH_4_N_2_O), Anhydrous ethanol, Tetraethylorthosilicate (TEOS), Cetyltrimethylammonium bromide (CTAB), N,N-Dimethylformamide (DMF), (3-dimethylaminopropyl)ethyl-carbodiimidmonohydrochloride (EDC), Beta-cyclodextrin (β-CD), Fluorescein isothiocyanate (FITC), Dexamethasone (DEX), γ-Oxo-1-pyrenebutyricacid (Py), and 3-Aminopropyltriethoxysilane (ATPES) were purchased from Aladdin Biochemical Technology Co. Ltd. Concentrated nitric acid, Ammonium Hydroxide (NH_3_·H_2_O), and Acetone were bought from Sinopharm Chemical Reagent Co. Ltd. All reagents were analytical grade, and the deionized water was obtained by ultrapure water mechanism (Vent Filter MPK01, Merck Millipore).

Cell Counting Kit-8 was ordered from Beyotime. Mounting Medium, antifading (with DAPI), Calcein-AM/PI live cell/dead cell double staining kit, Triton X-100, Hematoxylin-Eosin/HE Staining Kit were obtained from Solarbio Life Sciences. Enzyme-linked immunosorbent assay (ELISA) kits for the detection of inflammatory factors (IL-1β, IL-6 and TNF-α) were obtained from Elabscience Biotechnology. Dulbecco’s Modified Eagle Medium (DMEM, with or without high glucose, L-glutamine, phenol red, sodium pyruvate), Fetal bovine serum (FBS), were purchased from Gibco. RAW264.7, mouse mononuclear macrophage leukemia cells, tissue source of male Abelson mouse leukemia virus-induced tumors, were purchased from Procell Life Science&Technology Co. Ltd.

C57BL/6 mice, clean grade, 7–9 weeks old, weighing about 20–25 g, were purchased from the Experimental Animal Center of Guangxi Medical University. The mice were housed in an SPF-grade animal laboratory, with normal feeding, free access to water, and an ambient temperature of about 23°C. All animal protocols conformed to the animal guidelines of the Institutional Animal Care and Use Committee of Guangxi Medical University.

### 2.2 Characterization

Transmission electron microscopy (TEM) images were observed on an electron microscope (JEM-3010, JEOL). UV/Vis spectra were acquired with a UV/Vis spectrophotometer (UV-2600, Shimadzu). Fourier transform infrared (FT-IR) spectrum was recorded on a FT-IR spectrometer (Nicolet iS10, Thermo Fisher Scientific). Cell viability was obtained by a microplate reader (Infinite M Plex, Tecan). Fluorescence microscopy results were evaluated by fluorescence microscopy (LSM 980, ZEISS).

### 2.3 Synthesis of Y(OH)_*x*_(CO_3_)_*y*_F_*z*_:Yb/Tm@Y(OH)_*x*_(CO_3_)_*y*_F_*z*_:Yb precursors

Y_2_O_3_ (0.695 mmol), Yb_2_O_3_ (0.30 mmol), Tm_2_O_3_ (0.005 mmol), nitric acid, and ultrapure water were prepared for rare earth nitrate solution. Then, 1 ml of Y(NO_3_)_3_, 1 ml of Yb(NO_3_)_3_, 1 ml of Tm(NO_3_)_3_, 0.0581 g (1 mmol) of KF, and 3 g (0.05 mmol) of urea were added to a beaker. The beaker was volume fixed with ultrapure water to 50 ml and stirred for 5 min until the powder was completely dissolved. The beaker was placed in a constant temperature water bath and maintained at 90°C for 3 h. After the co-precipitation reaction, white flocculent precipitates were obtained at the bottom of the vessel. Then the beaker was taken out and cooled to room temperature. Afterwards, the precipitates were thoroughly cleaned 3 times with ultra-pure water and anhydrous ethanol, respectively. After drying overnight, the precursor Y(OH)_*x*_(CO_3_)_*y*_F_*z*_:Yb/Tm@Y(OH)_*x*_(CO_3_)_*y*_F_*z*_:Yb was finally obtained.

### 2.4 Synthesis of YOF:Yb/Tm@YOF:Yb@mSiO_2_ (UCNPs@mSiO_2_)

The precursor polymer was calcined at 700°C for 3 h to obtain YOF:Yb/Tm@YOF:Yb (UCNPs) for particle size analysis and long-range stability experiments. 0.2 g of precursor polymer, diluted with 50 ml absolute ethanol and 70 ml ultra-pure water. Add 0.3 g of cetyltrimethylammonium bromide (CTAB), 300 μL of ethyl orthosilicate (TEOS), and 1 ml of concentrated ammonia to the mixture in turn. The mixture was stirred until the substance was completely dissolved, followed by magnetic stirring at 25°C for 6 h. After centrifugation, the precipitates were cleaned with ultrapure water and anhydrous ethanol. Subsequently, the dried product was calcined at 700°C for 3 h to produce the core-shell structure product UCNPs@mSiO_2_.

### 2.5 Synthesis of UCNPs@mSiO_2_-Py

1.8 g of UCNPs@mSiO_2_ was dissolved in 20 ml of NaOH solution (1 M), stirred continuously for 8 h, and dried in an oven (60 °C) overnight. Afterwards, the products were dispersed in a mixed solution of 70 ml deionized water and 20 ml APTES and stirred for 8 h at 55°C. The precipitate was gathered by centrifugation, and the precipitate was alternately cleaned with anhydrous ethanol and ultrapure water for 3 times. Finally, the product UCNPs@mSiO_2_-NH_2_ was obtained after drying at 60°C for 12 h. UCNPs@mSiO_2_-NH_2_ was added to an ethanol solution containing Py (10 mM) and EDC (50 mM), then stirred for 12 h at 50°C. The precipitation was recovered by centrifugation and washed thoroughly with ethanol to attained UCNPs@mSiO_2_-Py.

### 2.6 UCNPs@mSiO_2_-Py was loaded with DEX and modified by FITC-β-CD

50 mg β-CD was dissolved in 5 ml of anhydrous DMF, followed by adding 0.3 mM fluorescein isothiocyanate (FITC) and stirring at 30°C for 12 h. Acetone was added to the above solution to precipitate the precipitate and then precipitated by ethanol solution. After acetone was added, FITC/β-CD was collected by centrifugation and thoroughly cleaned with anhydrous ethanol.

DEX solution (10 ml, 1 mM) was added to a beaker containing UCNPs@mSiO_2_-Py (100 mg). The mixture was dispersed by ultrasonic for 30 min and stirred continuously for 24 h to diffuse the drug into the nanopores of UCNPs@mSiO_2_-Py. After that, the precipitates UCNPs@mSiO_2_(DEX)-Py and supernatant were respectively gathered by centrifugation. Then, UCNPs@mSiO_2_(DEX)-Py and FITC/β-CD (1 g) were added to a beaker and stirred continuously for 12 h. The precipitates UCNPs@mSiO_2_(DEX)-Py/β-CD/FITC (USDPFs) were recovered by centrifugation, purified with ultrapure water, and dried in a drying oven. The amount of DEX in the supernatant was converted from the UV-Vis spectrum of DEX (absorbance at 291 nm).

### 2.7 DEX loading and releasing tests in USDPFs

DEX was dissolved in ultrapure water and diluted into different concentration gradients (500, 250, 125, 62.5, 31.25, 15.6, 0 μg ml^−1^). The absorbance corresponding to each concentration gradient at 240 nm wavelength was detected with a UV spectrophotometer, and a standard curve for DEX was drawn.

Aspirate the supernatant in step 2.6, dilute it with ultrapure water, use an ultraviolet spectrophotometer to detect the absorbance at the wavelength of 240 nm, and calculate the concentration of free DEX in the supernatant according to the standard curve of DEX: Drug loading rate (%) = (Amount of drug added - The amount of drug in the supernatant)/Amount of nanocarriers. An appropriate amount of USDPFs was made into a suspension in ultrapure water. After irradiating it with 980 nm NIR for different gradient time, the absorbance at 240 nm wavelength was detected by ultraviolet spectrophotometer (UV/Vis spectrometer). Similarly, the content of DEX in the supernatant was figured out based on the standard curve of DEX, and the DEX release curve with the change of NIR irradiation time was drawn.

### 2.8 Biocompatibility of USDPFs evaluation

RAW264.7 cells with good growth status were incubated into 96∼ well plates (seeding density: 2×10^3^ cells/well) and cultured in an incubator (37°C, 5% CO_2_) for 4 h. The cell culture medium in the corresponding wells was replaced with the medium containing different concentrations of USDPFs, so that the final concentration of USDPFs in each well plate decreased gradually (500, 250, 125, 62.5, 31.25, 15.625, 7.8125 μg ml^−1^) and incubated for 24 h. Then, CCK-8 solution (10 μL per well) was added, and the cells were continued to be cultured for 3 h. Use a microtiter plate reader to read the absorbance (OD value) of each group of samples at 450 nm.

### 2.9 Hemolysis assay of USDPFs

Human whole blood was purified by centrifugation and washed with 1% normal saline several times to obtain red blood cells. Dilute red blood cells with PBS solution (pH 7.4), and add 200 μL USDPFs solution prepared in PBS solution, with a total volume of 4 ml. The concentrations of USDPFs were 7.8125 μg ml^−1^, 15.625 μg ml^−1^, 31.25 μg ml^−1^, 62.5 μg ml^−1^, 125 μg ml^−1^, 250 μg ml^−1^, and 500 μg ml^−1^, respectively. The erythrocytes were cultured with USDPFs for 2 h at 37°C and centrifuged to obtain the supernatant. Then, read the absorbance of the supernatant of each group of samples at 541 nm. The equation Hemolysis % = (OD_sample_ − OD_control(−)_)/(OD_control(+)_ − OD_control(−)_) was employed on calculating the percent hemolysis of red blood cells, where OD presents the absorbance values of supernatants at 541 nm, OD_control(−)_ means the negative control (RBC dissolved in PBS solution), and OD_control(+)_ means the positive control (RBCs dissolved in ultrapure H_2_O).

### 2.10 Cellular uptake of USDPFs sample

UCNPs, UCNPs@mSiO_2_, and USDPFs were prepared with DMEM medium at a concentration of 50 μg ml^−1^. RAW264.7 cells were co-cultured with these three solutions for 6 h. Then the cells were washed with PBS solution, labeled by adding DAPI fluorescent dye and observed under a confocal microscope.

### 2.11 *In vivo* safety evaluation of USDPFs

Normal saline and USDPFs (5 mg kg^−1^) was injected into healthy male C57BL/6 mice by intratracheal administration, and the mice were sacrificed 2 weeks later. The main organs (heart, lung, liver, spleen, kidney) were collected for hematoxylin and eosin (H&E) staining. USDPFs (5 mg kg^−1^) was injected into mice through intratracheal administration. After 1 day, 3 days, 7 days, 14 days, and 21 days, the mice were euthanized and lung tissues were collected for H&E staining. The morphological changes of organ tissues were observed under an optical microscope. Inject USDPFs (5 mg kg^−1^) into the tail vein of mice for pharmacokinetic testing.

### 2.12 *In vivo* imaging effects of USDPFs

Normal saline, USDPFs (5 mg kg^−1^) were injected into healthy C57BL/6 mice by intratracheal administration. *In vivo* fluorescence imaging of USDPFs was recorded by taking pictures of mice with a small animal imager.

### 2.13 Anti-inflammatory effect of USDPFs in LIRI *in vitro* model

Referring to previous research (Wang et al., 2020), an oxygen-glucose deprivation and reperfusion (OGD/R) model was developed to simulate *in vitro* lung I/R. RAW264.7 cells were divided into a control group, USDPFs group, USDPFs + NIR group, OGD/R group, OGD/R + USDPFs group, and OGD/R + USDPFs + NIR group. OGD/R group: cells were incubated in a Whitley H35 Hypoxystation containing 1% O_2_, 5% CO_2_, and 94% N_2_ at 37°C (Don Whitley Scientific, Bingley, UK) for 1 h. The used medium was then removed and a medium containing serum, high sugar and no antibiotics was added. Cells were continued to be cultured in a normal incubator for 6 h. Cells in all groups were precultured with USDPFs (50 μg ml^−1^) for 6 h in addition to the control and OGD/R groups. After that, cells in the USDPFs + NIR group were irradiated with 980 nm NIR (10 min, 0.5 W cm^−2^), and cells in the OGD/R + USDPFs + NIR group were irradiated with 980 nm NIR (10 min, 0.5 W cm^−2^) after OGD/R. Biological transmission electron microscopy (Bio-TEM) of RAW264.7 Cells.

RAW264.7 cells in each group were collected, fixed with glutaraldehyde (3%) overnight and osmic acid (1%) for 2 h. The cells were subsequently dehydrated with acetone and embedded in resin. The samples were cut into ultra-thin slices and observed under a Bio-TEM (Hitachi H-7560, Tokyo, Japan).

#### 2.13.1 Live/dead cell staining

RAW264.7 cells were seeded in 24-well plates (seeding density: 1×10^5^ cells/well) and cultured for 4 h. Incubation was continued for 6 h after the addition of USDPFs (50 μg ml^−1^). Then, the USDPFs that were not taken up by the cells were removed, and the cells were cultured for 24 h. After washing the cells three times with PBS buffer, add 50 μL of FDA (labeled live cells, green fluorescence, 8 μg ml^−1^) and PI (labeled dead cells, red fluorescence, 20 μg ml^−1^ in PBS buffer to the cells) for 5 min. Discard the staining solution, add freshly prepared PBS buffer, and observe the cells under a fluorescence microscope.

#### 2.13.2 Detection of inflammatory factors in cell supernatant by ELISA

The cell supernatants of each group were retained, and the expression of inflammatory factors (IL-1β, IL-6 and TNF-α) in the cell supernatant was measured separately by ELISA kits.

### 2.14 Anti-inflammatory effect of USDPFs in LIRI *in vivo* model

Thirty healthy clean-grade male C57BL/6 mice at 7–9 weeks, weighing about 20–25 g, were randomly divided into 6 groups (n = 5): Control group, USDPFs group, USDPFs + NIR group, I/R + USDPFs group, I/R + USDPFs + NIR group. Ischemia/reperfusion (I/R) group (based on a previous study (Liang et al., 2021)): the chest cavity was opened from the left chest of mice, and the left hilum was clamped for 1 h by a non-invasive vascular clamp (maintaining the state of ischemia and hypoxia). Then released the hemostatic clamp, opened the left hilum, and reperfusion for 6 h. Except for the control group and the I/R group, the mice in the other groups were given USDPFs suspension (5 mg kg^−1^) by intratracheal injection 1 h before the operation. The mice in the USDPFs + NIR group were irradiated by 980 nm NIR (3 × 10 min, 1 W cm^−2^) after 2 h of administration. The mice in the I/R + USDPFs + NIR group were irradiated by 980 nm NIR (3 × 10 min, 1 W cm^−2^) after suturing the incision. After 6 h, mice were sacrificed by exsanguination. Serum and left lung tissue were collected. Similarly, after intratracheal injection of USDPFs (5 mg kg^−1^) and use of dexamethasone aerosol (DEX-A) in mice, pathological changes in lung tissue were observed.

#### 2.14.1 H&E staining

Lung tissue samples were fixed with 4% paraformaldehyde, embedded in paraffin, sectioned, stained with H&E, and explored with a fluorescence microscope. Lung tissue damage was scored as described below, based on the accumulation or infiltration of inflammatory cells in the vessel wall or in the air spaces [1 = vessel wall only, 2 = small amount in alveolar space, 3 = moderate amount, 4 = severe (alveolar space congestion)], presenting alveolar hyaline and interstitial congestion [1 = normal, 2 = mild (>25% of the field), 3 = moderate (25–50% of the field) and 4 = severe (>50% of the field)], and with (1) or without (0) hemorrhage (Murray and Wynn, 2011). The scores of the above items were added to obtain the lung injury score of the sample.

#### 2.14.2 Wet to dry weight ratio

The lung wet-to-dry (W/D) ratio was recorded to assess the degree of pulmonary edema. Fresh left lung tissue was gathered and weighed as wet weight instantly. Then, lung tissue samples were dried in a desiccator at 60°C for 48 h to a constant weight, recorded as dry weight, and the W/D ratio was calculated.

#### 2.14.3 Detection of inflammatory factors in serum and lung tissue by ELISA

Lung tissue was prepared as a tissue homogenate and serum was extracted from whole blood. The expressions of IL-1β, IL-6 and TNF-α in serum and lung tissue were detected by ELISA kits, respectively.

### 2.15 Statistical analysis

All data were dealt with the average of three independent experiments ± the standard deviation of the mean, Student’s t-test or one-way analysis of variance (SPSS 22.0), which could be employed on analysing the significant difference between the means of the groups was statistically significant. Statistically significant differences existed between groups when ^*^ *p* < 0.05.

## 3 Results and discussion

### 3.1 Preparation and analysis of the upconversion nanoparticles

**FIGURE 1:**
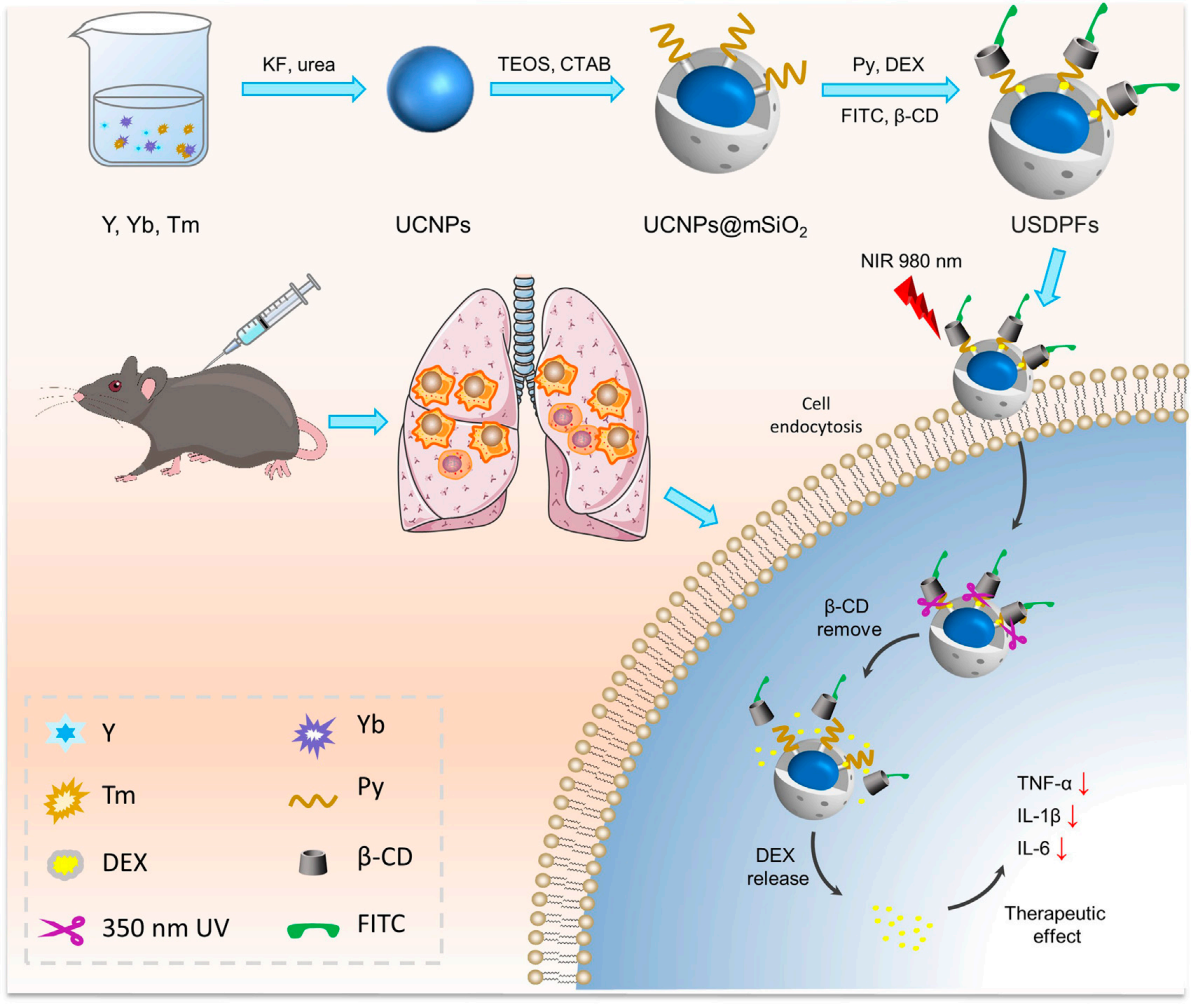
Schematic synthesis process and theranostic mechanism of nano-drugs for LIRI comprehensive therapy.

**FIGURE 2:**
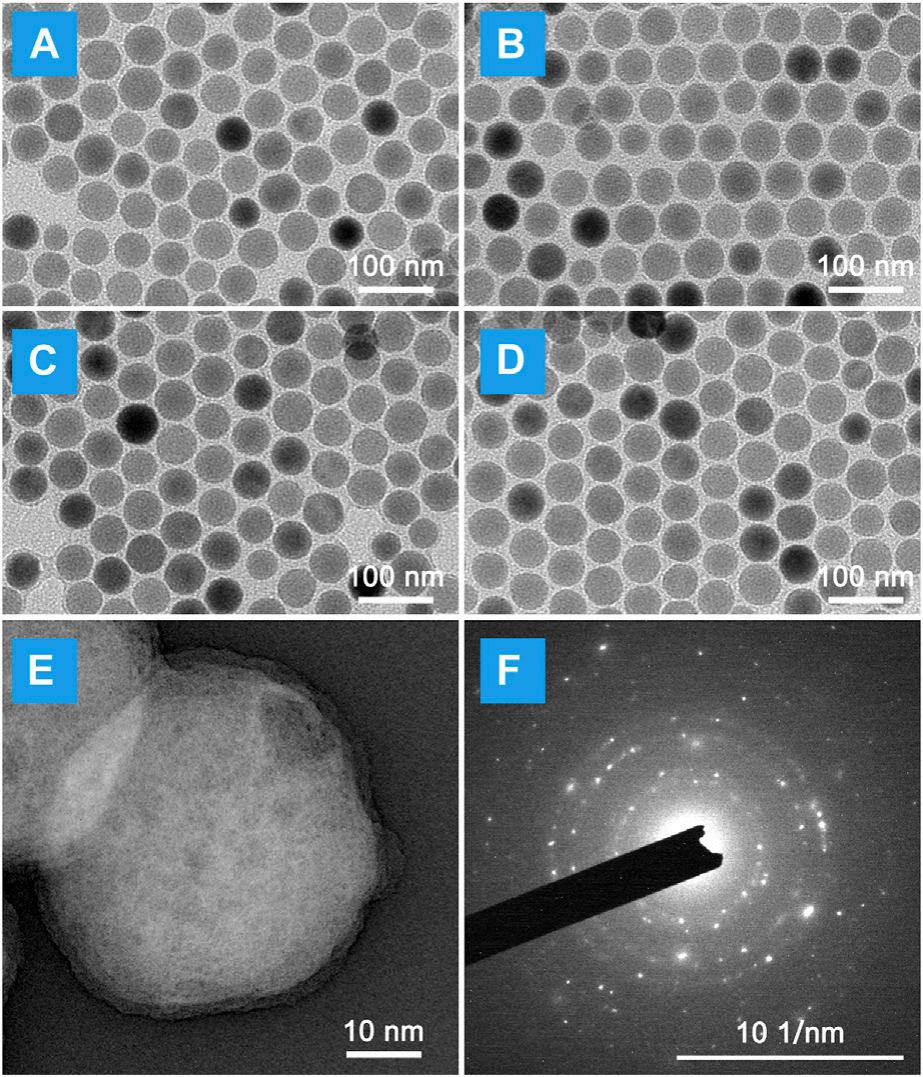
Micromorphology of LIRI comprehensive therapeutic nano-drugs at each synthesis process. TEM images of **(A)** UCNPs; **(B)** UCNPs@mSiO_2_; **(C)** UCNPs@mSiO_2_-Py; **(D)** UCNPs@mSiO_2_(DEX)-Py/β-CD/FITC (USDPFs). **(E)** STEM image and **(F)** Selected area electron diffraction (SAED) of USDPFs.

The schematic of NIR laser-irradiated upconversion nanoparticles with DEX precise released for LIRI comprehensive therapy was shown in Figure 1. It can be interpreted that the key to the precise release of DEX *in vivo* depends on the design and synthesis of nano-drugs. As illustrated in Supplementary Figure S1, UCNPs have a uniform particle size distribution and good stability, and do not decompose and modify over a long resting period. As images observed in Figures 2A, B, there was no obvious change in morphology, although particle size increased slightly (∼10 nm) after UCNPs coated mSiO_2_, indicating that mSiO_2_ layer was successfully tightly attached to UCNPs core. In Figures 2C, D, amorphous matter is obviously present on the surface of nanoparticles, which verifies that inorganic UCNPs@mSiO_2_ can be wrapped by polymer layers. The resulting nanohybrid UCNPs@mSiO_2_(DEX)-Py/β-CD/FITC (USDPFs) has a spherical morphology with a size of about 45–50 nm, uniform size and good monodispersity. In order to further investigate its crystallographic properties, STEM and SAED were measured simultaneously (Figures 2E, F). The results exhibited that the cladding structure was symmetry and the crystallinity was excellent.

**FIGURE 3:**
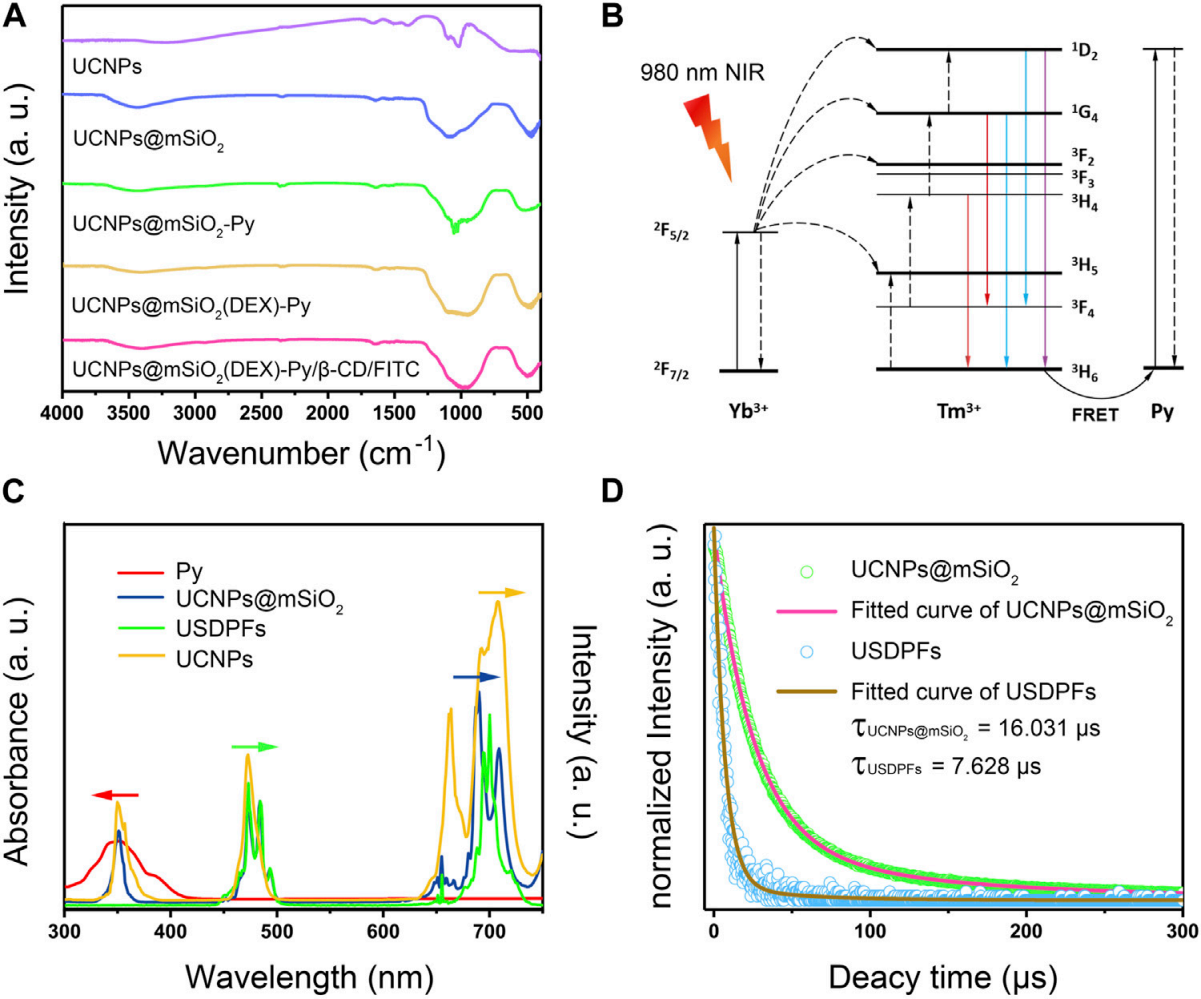
**(A)** FT-IR spectra of specimens in different synthesis steps; **(B)** Energy transfer mechanism between upconversion nanoparticles and light-responsive molecule Py; **(C)** UV-Vis absorption spectra of Py overlapped fluorescence emission spectra of UCNPs, UCNPs@mSiO_2_ and USDPFs; **(D)** Fluorescence lifetime curve of UCNPs@mSiO_2_ and USDPFs at 695 nm.

Figure 3A proves the changes of functional groups on the surface of nanoparticles at different synthesis stages, indicating the successful coating and accurate synthesis of layer, which is consistent with the TEM results. The excellent coating effect provides a close enough distance for the interlayer fluorescence resonance energy transfer, energy level transition process was designed in Figure 3B. Yb^3+^ ions in UCNPs absorb 980 nm light and transmit photons to Er^3+^ ions for visible light emission. Meanwhile, by collaborating Förster resonance energy transfer (FRET) effect, some of the blue emission light is absorbed by Py, which can break the ester bond adjoining the pyrene chromophore bond. As explored in Figure 3C, the strongest emission of blue and red light is at 475 nm and 695 nm, corresponding to ^1^G_4_→^3^H_6_ and ^1^G_4_→^3^F_4_ transition emissions separately. (Pengpeng et al., 2019). Since Py is strongly absorbed around 350 nm, the absorption peak of USDPFs relative to UCNPs@mSiO_2_ disappears in blue light emissions. Also, the visible emission intensity of fluorescence decreased slightly due to the increase of layer thickness on the nanohybrids. Moreover, the fluorescence lifetime of USDPFs was less than UCNPs@mSiO_2_, which was consistent with the results of fluorescence intensity.

### 3.2 DEX loading and release of USDPFs nano-drugs

**FIGURE 4:**
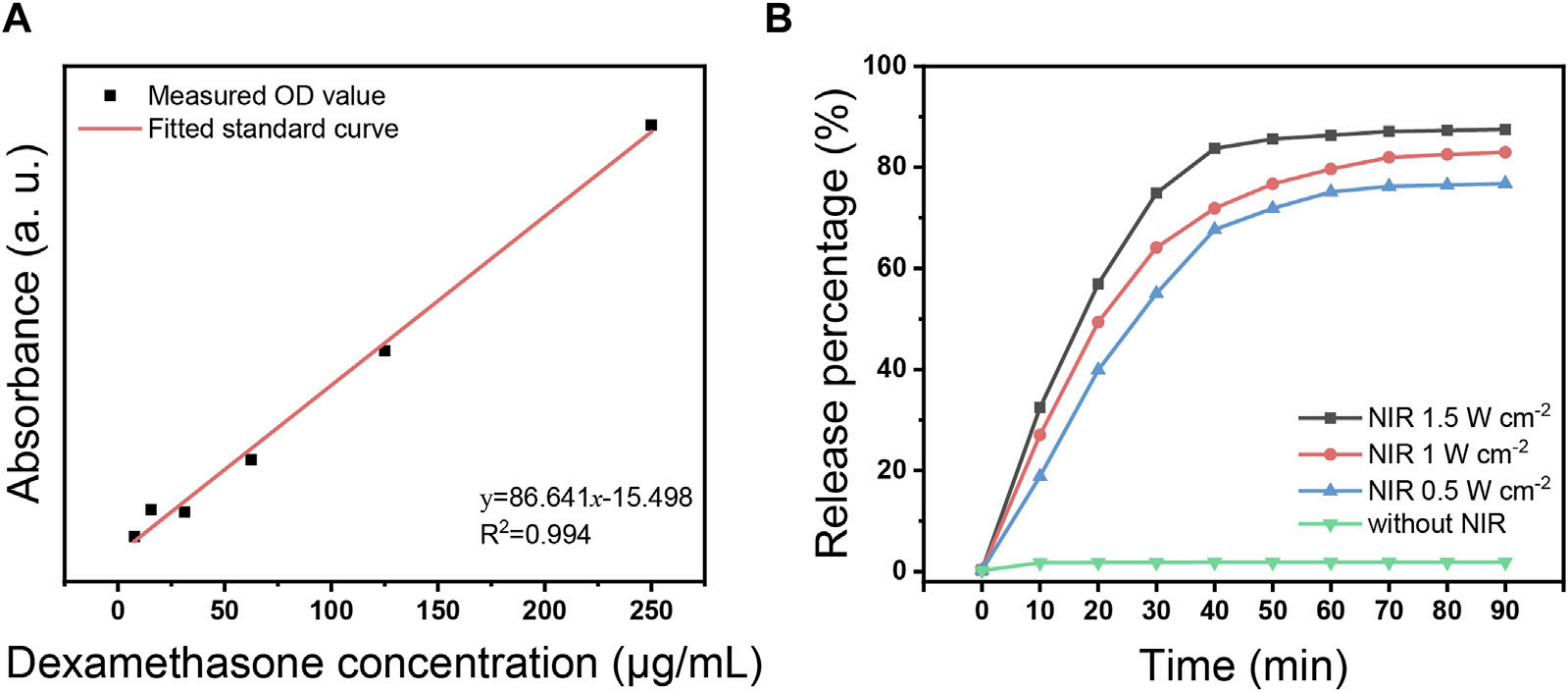
**(A)** Fitted standard curve of DEX in different concentrations; **(B)** DEX release curve with time under 980 nm NIR laser irradiation at different power.

Figure 4A indicates the fitted standard curve of absorbance at 240 nm for different concentration gradients of DEX. Based on the absorbance of free DEX released from USDPFs, the drug loading rate was calculated to be 11.46%.

Figure 4B demonstrates the release of DEX from USDPFs after NIR irradiation. In the absence of NIR irradiation, the amount of DEX in the solution was almost zero; after NIR initiation, the release of DEX gradually increased with increasing irradiation time and irradiation power. The increase in DEX concentration was most pronounced when the irradiation time accumulated to 30 min, at which point the amount released was more than half. After the irradiation time accumulated to 40 min, the release was close to the peak; the release was up to 87.5% when accumulated to 90 min.

### 3.3 *In vitro* evaluation of biocompatibility, hemolysis, and cellular uptake of USDPFs

**FIGURE 5:**
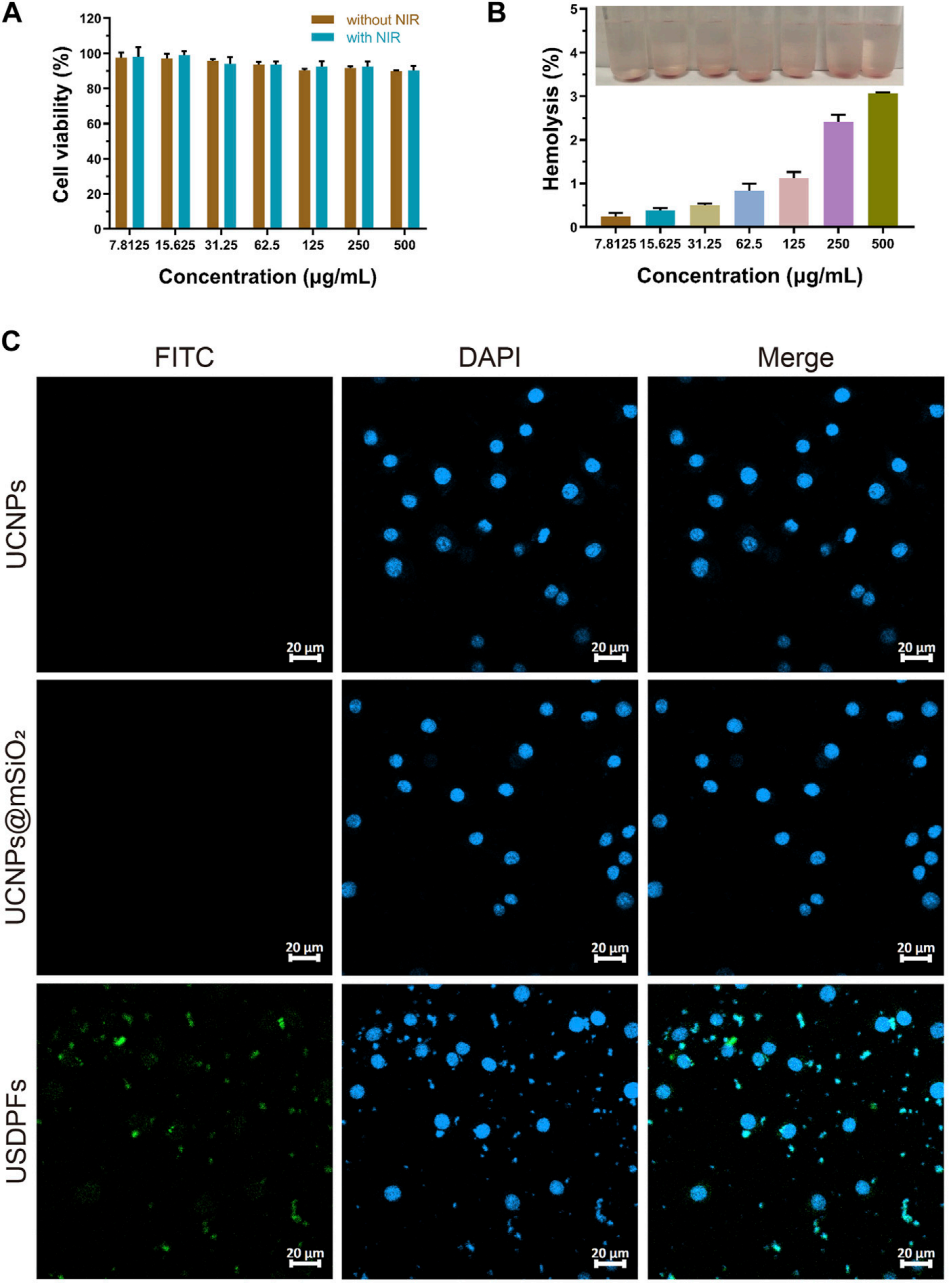
**(A)** Cell viability measurement of USDPFs with different concentrations; **(B)** Hemolysis test of USDPFs *in vitro*; **(C)** Cellular uptake of USDPFs (50 μg ml^−1^) under CLSM observation (× 400, scale bar 20 μm).

RAW264.7 cells were co-cultured with USDPFs and then subjected to CCK-8 assay to detect cell viability. The cell viability of each group decreased with increasing concentration of USDPFs, as explained in Figure 5A. To further test the biocompatibility of USDPFs, USDPFs were co-cultured with human erythrocytes for hemolysis assay. Figure 5B illustrates that the lysis of erythrocytes increased as the concentration of USDPFs increased. The above results indicate that the silica-coated and β-CD-modified USDPFs have high biosafety.

Figure 5C exhibits the results of the cellular uptake of USDPFs under confocal microscopy. DAPI stains the nuclei bright blue. A large number of USDPFs modified with FITC appear green under the microscope and surround the nucleus. Compared to UCNPs and UCNPs@mSiO_2_, the biggest difference of USDPFs is the presence of polymer coating (β-CD). The encapsulation of β-CD resulted in better water solubility, lower cytotoxicity, and better biocompatibility of the nanomaterials (Liu et al., 2019). Both USDPFs (encapsulated with β-CD) and cell membranes are organic components. Therefore, USDPFs are more compatible with cell membranes and facilitate cell phagocytosis. These results further indicate that USDPFs have good cytocompatibility and can be largely endocytosed by RAW264.7 cells for stable coexistence and fusion.

### 3.4 *In vivo* safety evaluation and prolonged blood circulation of USDPFs

**FIGURE 6:**
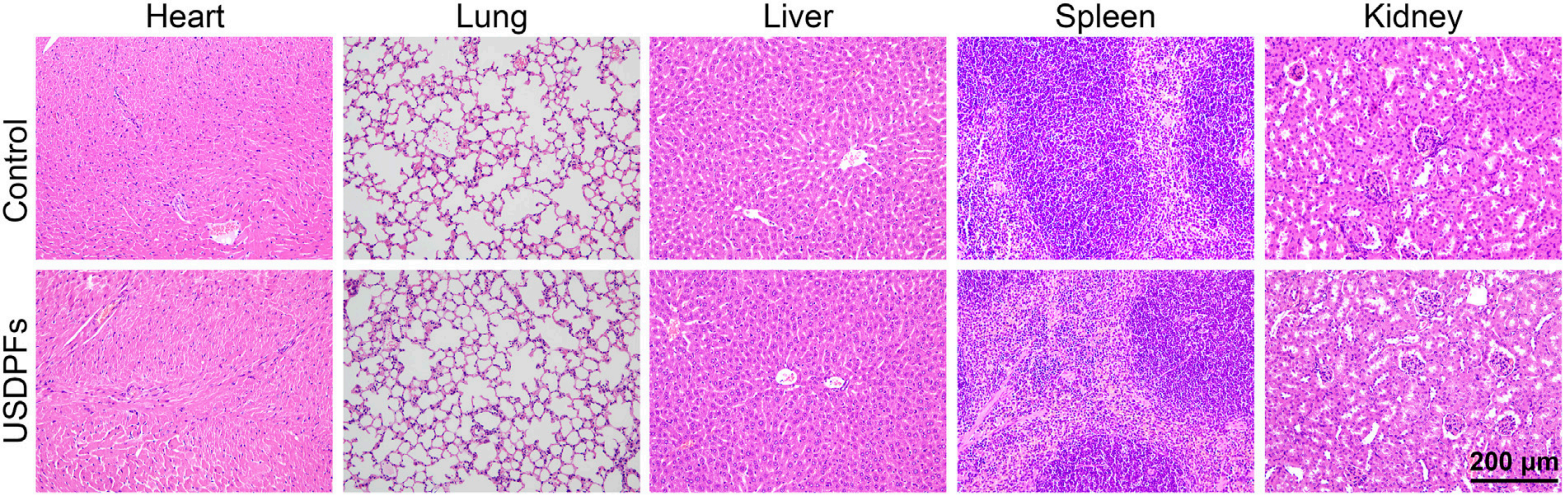
H&E staining of the heart, lung, liver, spleen and kidney of C57B/L6 mice 2 weeks after intratracheal injection of USDPFs (5 mg kg^−1^) (× 200, scale bar 200 μm).

To evaluate the safety of USDPFs *in vivo*, healthy adult C57B/L6 mice were injected intratracheally with USDPFs. After 2 weeks, the major organs (heart, lung, liver, spleen, and kidney) of mice were collected for H&E staining. As given in Figure 6, the mice in the USDPFs group did not have inflammatory cell infiltration and tissue necrosis in the heart, lung, liver, spleen, and kidney compared with the control group. As shown in Supplementary Figure S2, after intratracheal administration of USDPFs for 1 day, 3 days, 7 days, 14 days, and 21 days, no significant damage was observed in the lung tissue pathology of the mice, indicating that the intratracheal administration of nanoparticles does no damage to lung tissues. These results indicated that USDPFs were not significantly toxic to some vital organs of mice and had good *in vivo* safety.

According to the references (Hu et al., 2019; Hu et al., 2021; Lu et al., 2023), an *in vivo* pharmacokinetics study of USDPFs (5 mg kg^−1^) was conducted to demonstrate the prolonged blood circulation of the nanoplatform for exerting the enhanced permeability and retention (EPR) effect. As shown in Supplementary Figure S3, after intravenous injection, USDPFs remain for about 10 h and gradually decrease, indicating that the blood circulation time of USDPFs in the body is prolonged, exerting the EPR effect.

### 3.5 *In vivo* imaging evaluation of USDPFs

**FIGURE 7:**
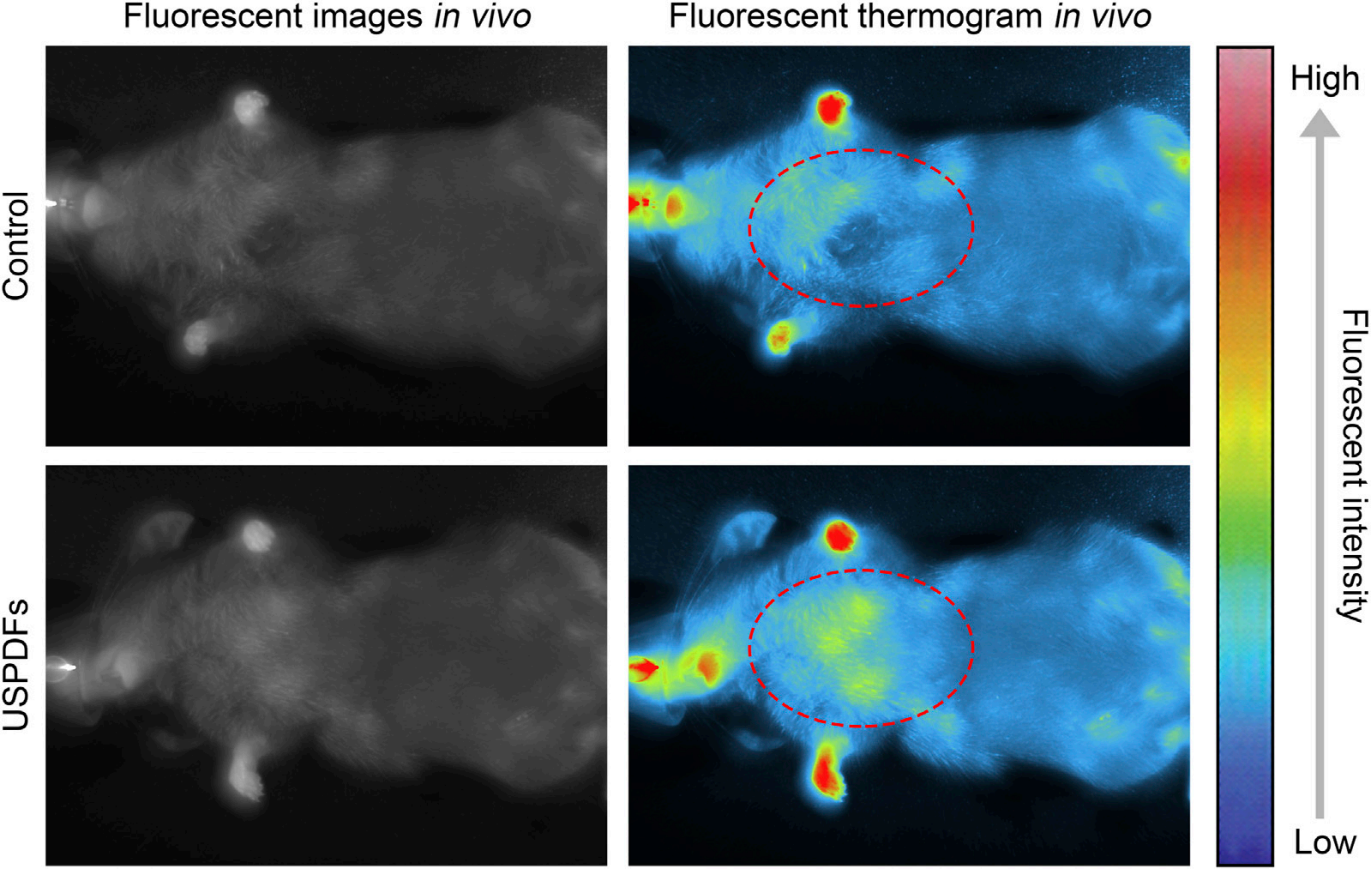
Small animal imager to observe the fluorescence imaging of USDPFs (5 mg kg^−1^) in mice after intratracheal administration.

Healthy adult C57B/L6 mice were intratracheally injected with USDPFs, and fluorescence imaging of USDPFs in mice was observed under 980 nm NIR irradiation (Figure 7). The fluorescence in mice appeared bright white, and USDPFs showed stronger fluorescence in mice compared to controls. This strong fluorescence contrast is more pronounced in thermogram images and is mainly concentrated in the lungs (marked by red dashed circles). These results suggest that USDPFs are mainly distributed in lung tissue after intratracheal injection and have effective *in vivo* imaging properties.

### 3.6 Evaluation of the *in vitro* anti-inflammatory effect of USDPFs

**FIGURE 8:**
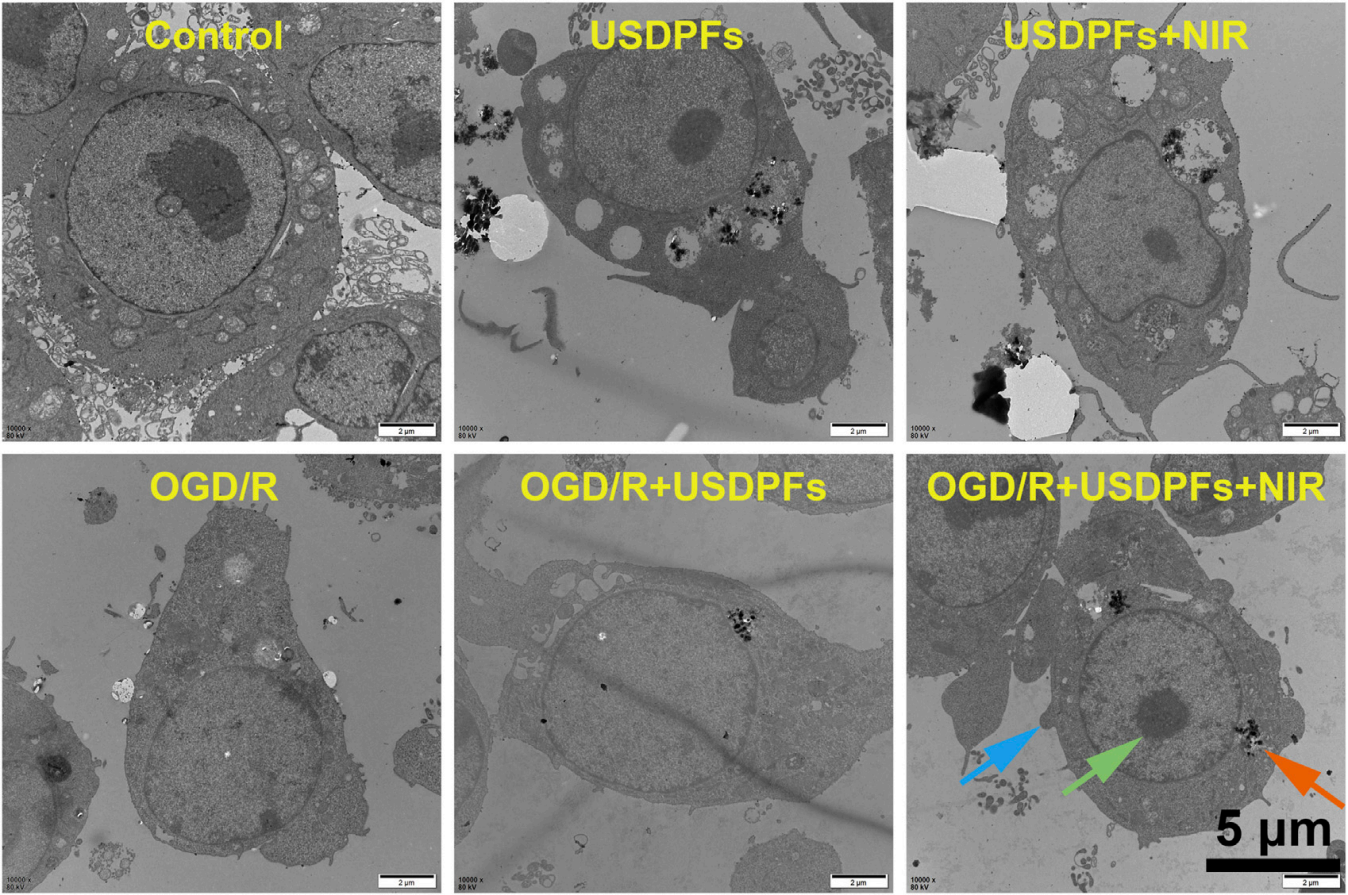
Ultrastructural changes of RAW264.7 cells in each group (×10000, scale bar 5 μm) (Cell processes are indicated by blue arrows, phagocytosis vesicles are indicated by orange arrows, and nucleoli are indicated by green arrows).

The ultrastructural changes of the cells in each group were detected by transmission electron microscopy (Figure 8). The control RAW264.7 cells apparently had a large number of protrusions on the cell membrane, intact intracellular organelles, and a large number of phagocytic vesicles. The ultrastructure of the ODG/R and OGD/R + USDPFs groups was severely damaged, with fewer protrusions or even disappearances. In addition, there were fewer phagocytic vesicles, reduced chromatin border set, and disappeared nucleoli. The cell damage in the OGD/R + USDPFs + NIR group was improved, and a small amount of organelles, such as phagocytic vesicles and nucleoli, were present.

**FIGURE 9:**
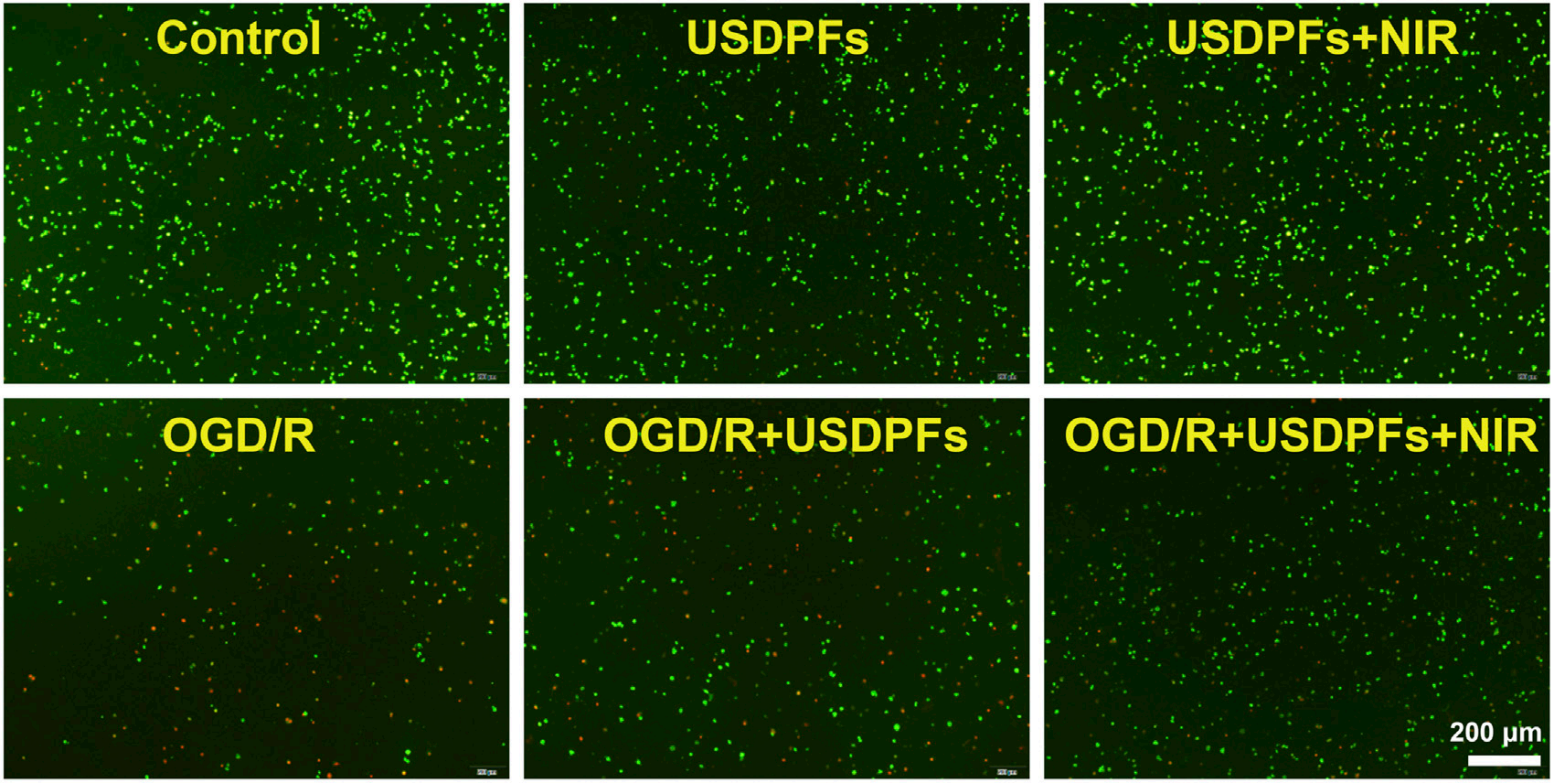
Cell viability/cytotoxicity detection of RAW264.7 cells in each group (×40, scale bar 200 μm) (Green fluorescence represents live cells, red fluorescence represents dead cells).

The apoptosis of the cells in each group was observed by double staining of live/dead cells. Figure 9 implies that the number of cells in the control, USDPFs, and USDPFs + NIR groups were essentially similar, with only a minimal number of apoptotic cells (stained in red). The difference is that the OGD/R and OGD/R + USDPFs groups have significantly fewer surviving cells (stained in green) and even a large number of apoptotic cells. The OGD/R + USDPFs + NIR group had more surviving cells and fewer apoptotic cells.

**FIGURE 10:**
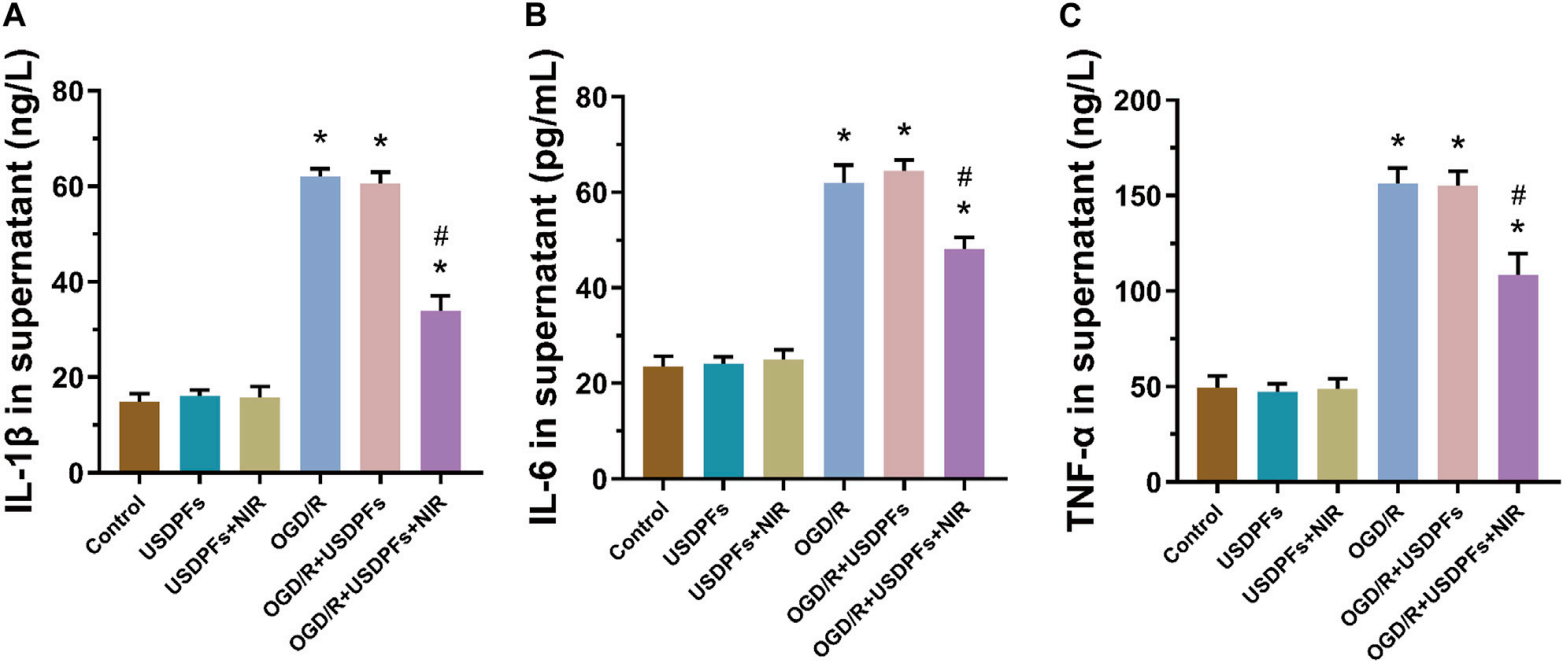
Expression of IL-1β **(A)**, IL-6 **(B)** and TNF-α **(C)** in the supernatant of RAW264.7 cells in each group (*n* = 5 per group,compared with the control group, ^*^ *p* < 0.05; Compared with the OGD/R group, ^#^ *p* < 0.05).

The expression of inflammatory mediators (IL-1β, IL-6 and TNF-α) in cell culture supernatants was measured by ELISA to assess the degree of inflammatory damage in each group of cells (Figure 10). First, there was no significant difference in cell damage in the USDPFs and USDPFs + NIR groups compared with the control group; cell damage was severe in both the OGD/R and OGD/R + USDPFs groups. Importantly, cellular inflammatory damage was significantly improved in the OGD/R + USDPFs + NIR group compared to the OGD/R and OGD/R + USDPFs groups.

The above results further suggest that NIR irradiation at 980 nm triggered the release of DEX, which ameliorated the cell damage induced by OGD/R.

### 3.7 Evaluation of the *in vivo* anti-inflammatory effect of USDPFs

**FIGURE 11:**
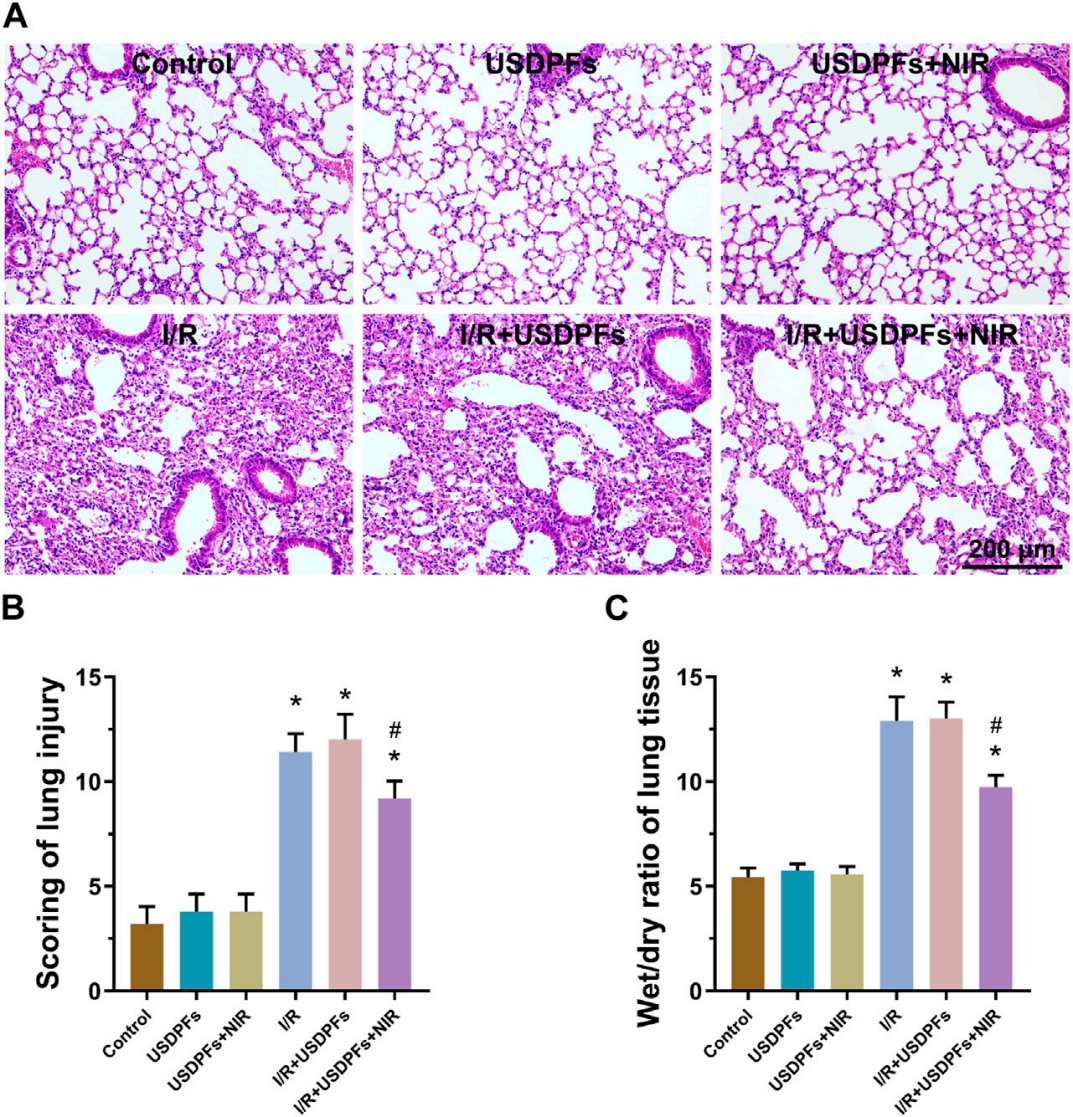
**(A)** H&E staining of lung tissue of mice in each group (×200, scale bar 200 μm); **(B)**. Pathological lung injury scores of mice in each group; **(C)**. Wet/dry ratio of lung tissue of mice in each group (*n* = 5 per group, compared with the control group, ^*^ *p* < 0.05; Compared with the I/R group, ^#^ *p* < 0.05).

Histopathological changes in C57B/L6 mice lung tissues in each group were observed under light microscopy. As explicated in Figure 11A, in control, USDPFs, and USDPFs+NIR groups, the lung tissue structure was clear and uniform in thickness, without congestion and inflammatory cell infiltration. In contrast, the alveolar walls of the I/R group and I/R+USDPFs group were thickened, with interstitial edema, and filled with a large number of erythrocytes and inflammatory cells. Compared with the I/R group, the I/R+USDPFs+NIR group had more apparent lung tissue contours, reduced edema, and fewer inflammatory cells and erythrocytes. The pathological changes after intratracheal injection of USDPFs (5 mg kg^−1^) and use of dexamethasone aerosol in mice are shown in Supplementary Figure S4. The effect of USDPFs is similar to that of dexamethasone aerosols, which can alleviate lung tissue damage after I/R.

Figure 11B illustrates the pathology score of lung injury. Compared to the control group, there was no significant difference between the USDPFs and USDPFs + NIR groups, and the scores of the I/R and I/R + USDPFs groups were significantly increased. Unexpectedly, the I/R + USDPFs + NIR group had significantly lower scores than the I/R and I/R + USDPFs groups.

The W/D values of the lung tissue represent the degree of pulmonary edema and inflammatory exudation. There was no difference in pulmonary edema between the USDPFs group and USDPFs + NIR group mice compared to the control group (Figure 11C). Encouragingly, the lung edema of mice in the I/R + USDPFs + NIR group improved compared to the I/R group and the I/R + USDPFs group.

**FIGURE 12:**
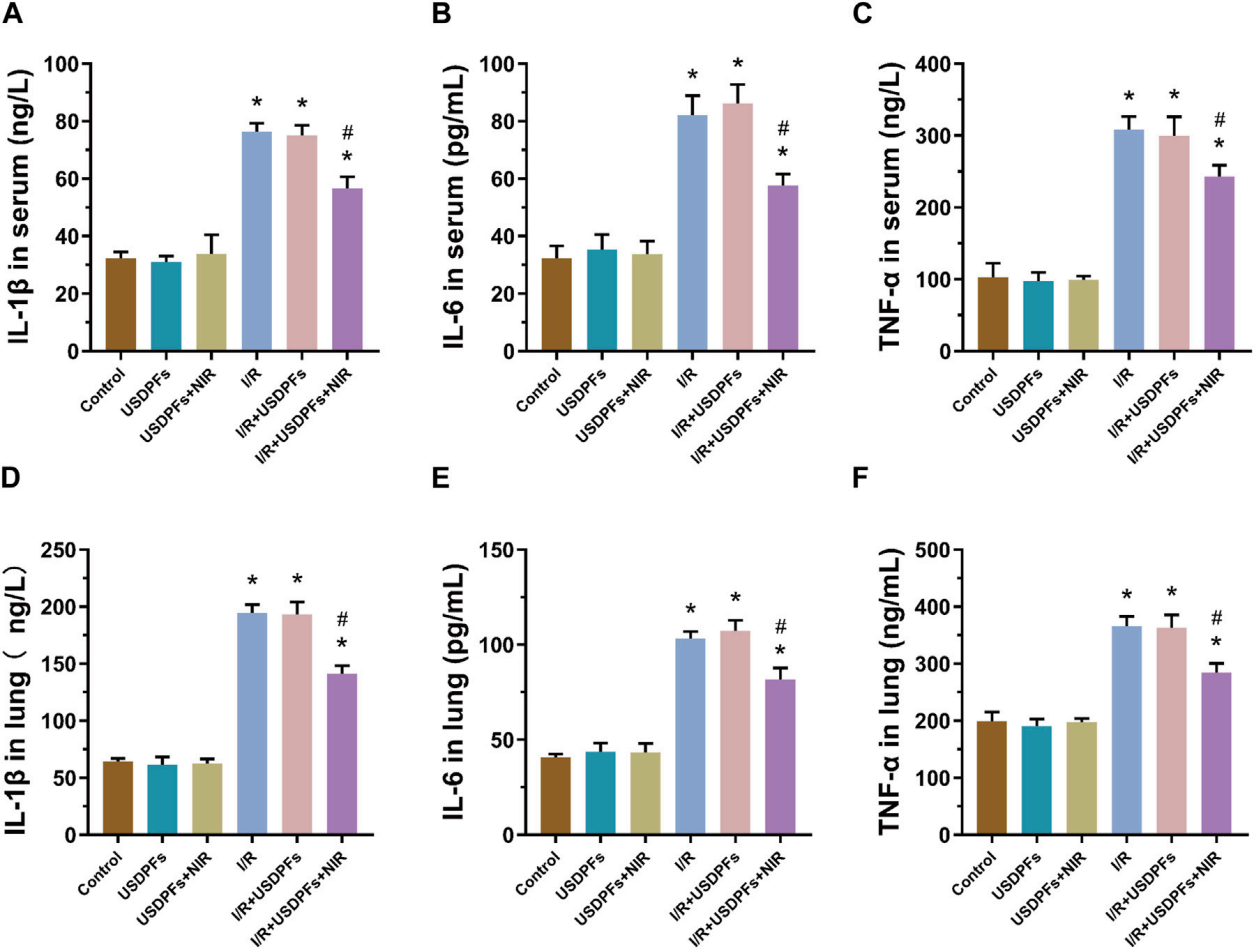
**(A-C)** Expression of IL-1β **(A)**, IL-6 **(B)** and TNF-α **(C)** in serum. **(D-F)** Expression of IL-1β **(D)**, IL-6 **(E)** and TNF-α **(F)** in lung tissue. (*n* = 5 per group, compared with the control group, ^*^ *p* < 0.05; Compared with the OGD/R group, ^#^ *p* < 0.05).

Inflammatory factors (IL-1β, IL-6 and TNF-α) were measured in mouse serum and lung tissue by ELISA to assess inflammatory damage in each group (Figure 12). Compared with the control group, the degree of inflammatory injury was not significantly different between the USDPFs and USDPFs+NIR groups of mice. In contrast, the inflammatory injury was more severe in mice’s I/R and I/R+USDPFs groups. Significantly, the mice in the I/R+USDPFs+NIR group had significantly lower inflammatory injury than those in the I/R and I/R+USDPFs groups.

The above results suggest that USDPFs has better biosafety, and 980 nm NIR irradiation triggers the release of DEX, which reduces the inflammatory damage and edema of lung tissue caused by I/R.

## 4 Conclusion

In this work, we construct a multi-functional upconversion nano-drug delivery system USDPFs for LIRI. First, DEX can be efficiently and accurately released from USDPFs in the intrapulmonary microenvironment after 980 nm laser irradiation, which can be directly applied to improve I/R-induced lung injury. Meanwhile, USDPFs have good upconversion fluorescence emission ability, biocompatibility, and hemolysis, supporting their excellent multiple fluorescence imaging performance *in vitro* and *vivo*.

## Data Availability

The original contributions presented in the study are included in the article/Supplementary Materials, further inquiries can be directed to the corresponding author.
