# Supplementary figures and images for: Near-infrared laser-irradiated upconversion nanoparticles with dexamethasone precise released for alleviating lung ischemia-reperfusion injury
Source: Front Bioeng Biotechnol. 2023 May 5;11:1176369. doi: 10.3389/fbioe.2023.1176369 (PMC10196198; doi:10.3389/fbioe.2023.1176369)

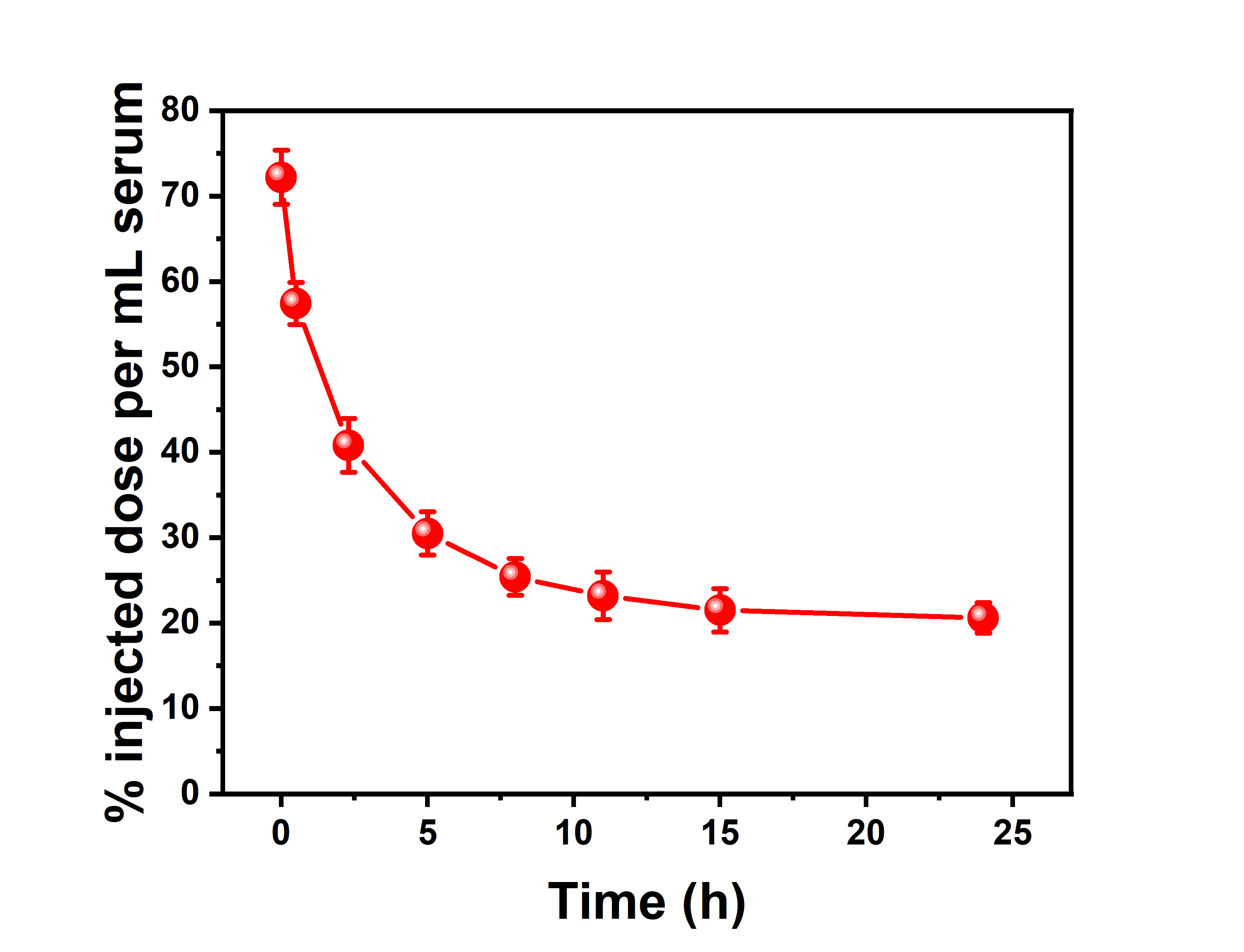

Supplement: Supplementary file 1 [file Image3.JPEG]

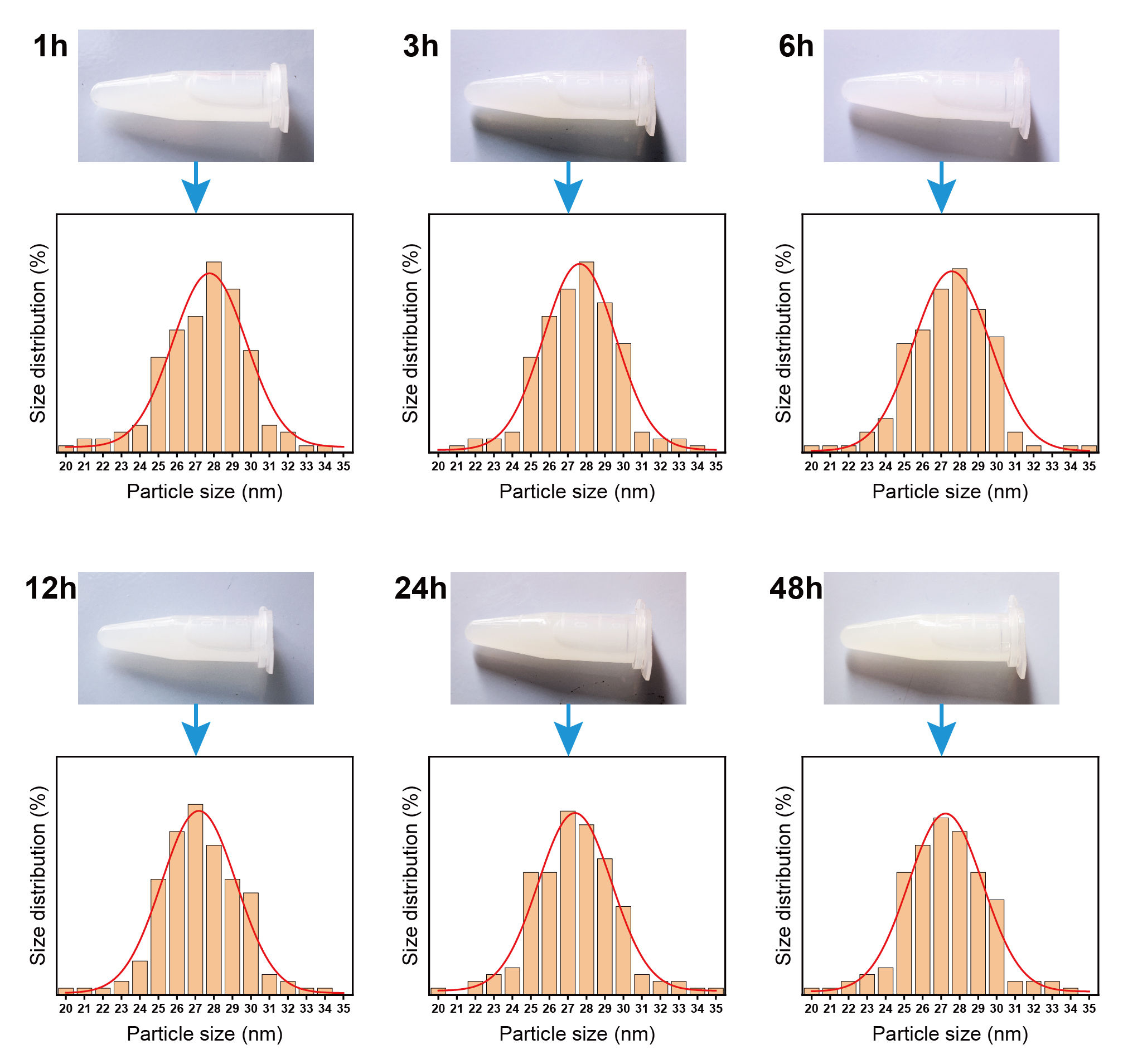

Supplement: Supplementary file 3 [file Image1.JPEG]

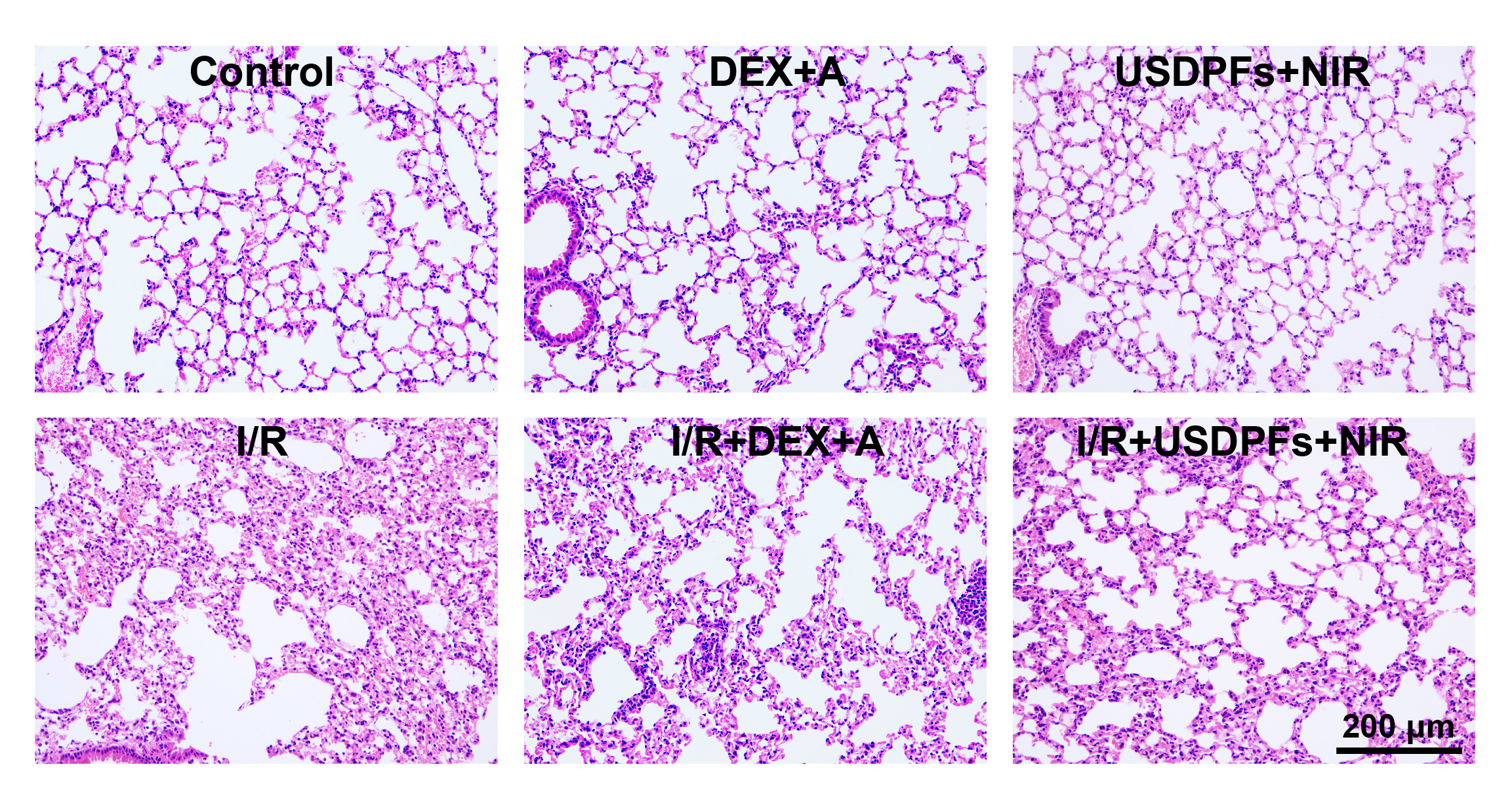

Supplement: Supplementary file 4 [file Image4.JPEG]

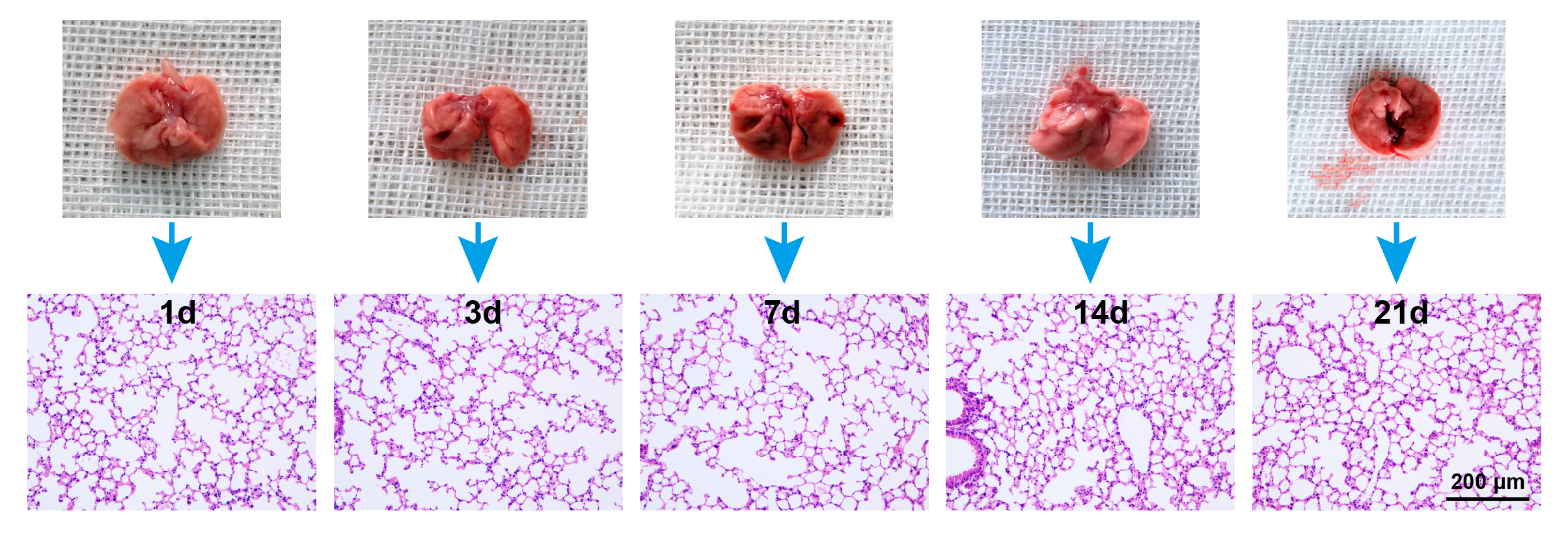

Supplement: Supplementary file 5 [file Image2.JPEG]
