# Supplementary material for: Near-infrared laser-irradiated upconversion nanoparticles with dexamethasone precise released for alleviating lung ischemia-reperfusion injury
Source: Front Bioeng Biotechnol. 2023 May 5;11:1176369. doi: 10.3389/fbioe.2023.1176369 (PMC10196198; doi:10.3389/fbioe.2023.1176369)
Supplement: Supplementary file 2 [file Table1.docx]

Supporting Information

Near-Infrared Laser-Irradiated Upconversion Nanoparticles with Dexamethasone Precise Released for Alleviating Lung Ischemia-Reperfusion Injury

**Xiaojing He^1,2,3,4^**^†^**, Zhining Li^1^**^†^**, Mengling Ye^1,3,4^, Chen Zhao^1,2,3,4^, Siyi Wu^1,2,3,4^, Yi Qin^1,2,3,4^, Youyuan Guo^1,2,3,4^, Lu Zhang^1,2,3,4^, Fei Lin^1,2,3,4*^**

^1^Guangxi Medical University Cancer Hospital, China, Nanning.

^2^Guangxi Clinical Research Center for Anesthesiology, China, Nanning.

^3^Guangxi Engineering Research Center for Tissue & Organ Injury and Repair Medicine, China, Nanning.

^4^Guangxi Key Laboratory for Basic Science and Prevention of Perioperative Organ Disfunction, China, Nanning.

^†^Xiaojing He and Zhining Li contributed equally to the research.

*** Correspondence:**

Fei Lin

E-mail: linfei@gxmu.edu.cn


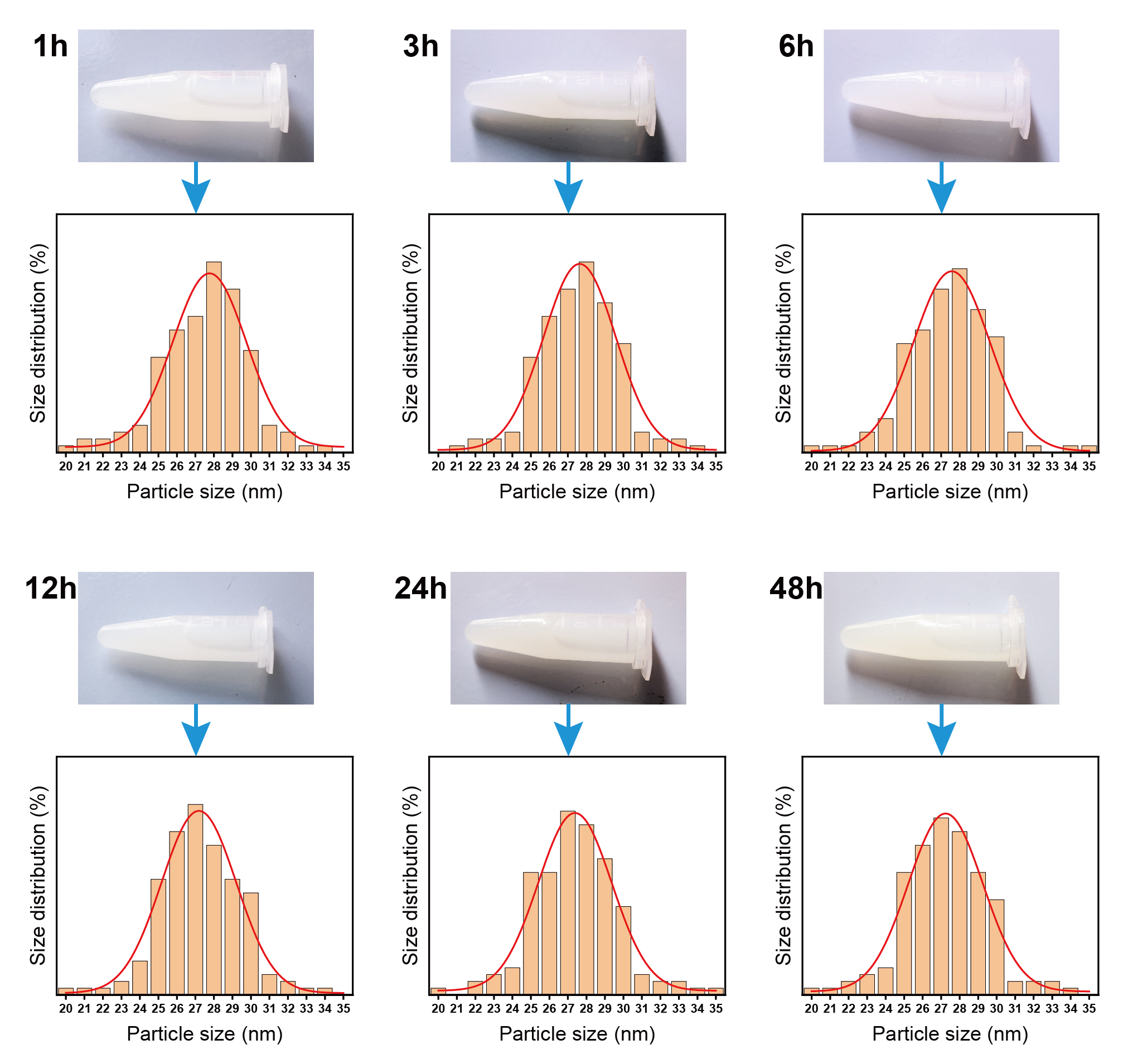


**Figure S1** The hydrodynamic stability and size of UCNPs under long-term storage (1h, 3h, 6h, 12h, 24h, 48h) test.


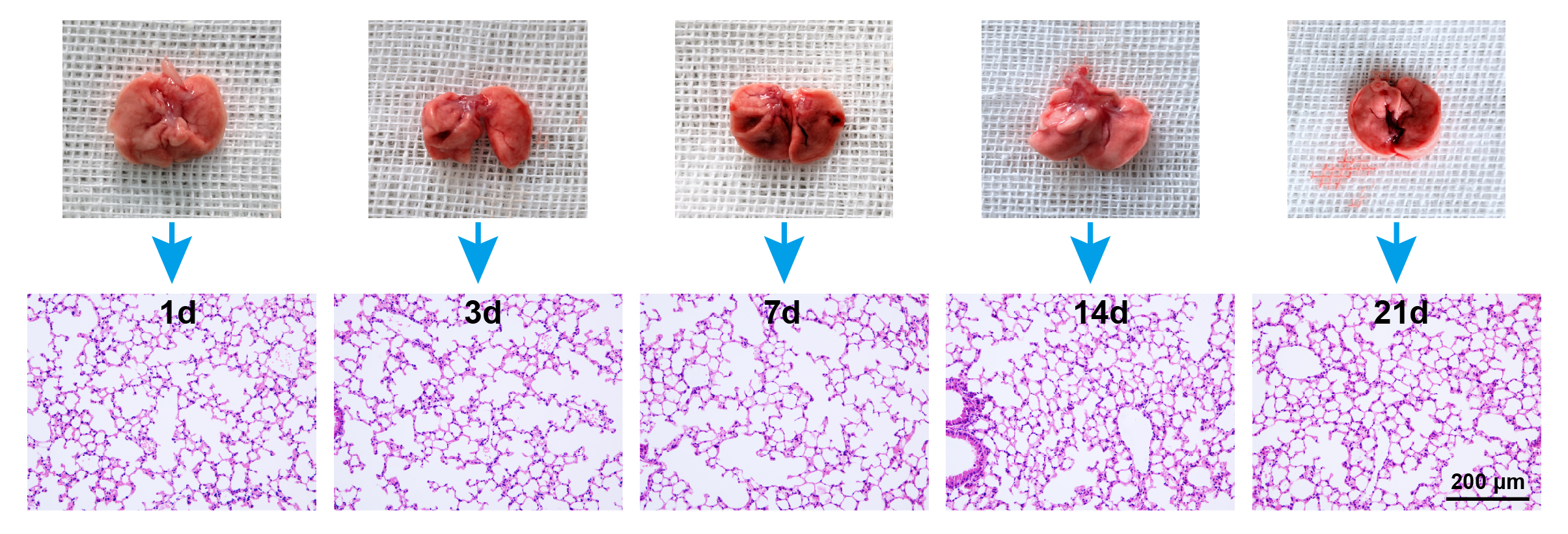


**Figure S2** H&E staining of lung tissue in mice after 1 day, 3 days, 7 days, 14 days, and 21 days of intratracheal administration of USDPFs (5 mg kg^-1^) (× 200, scale bar 200 μm).


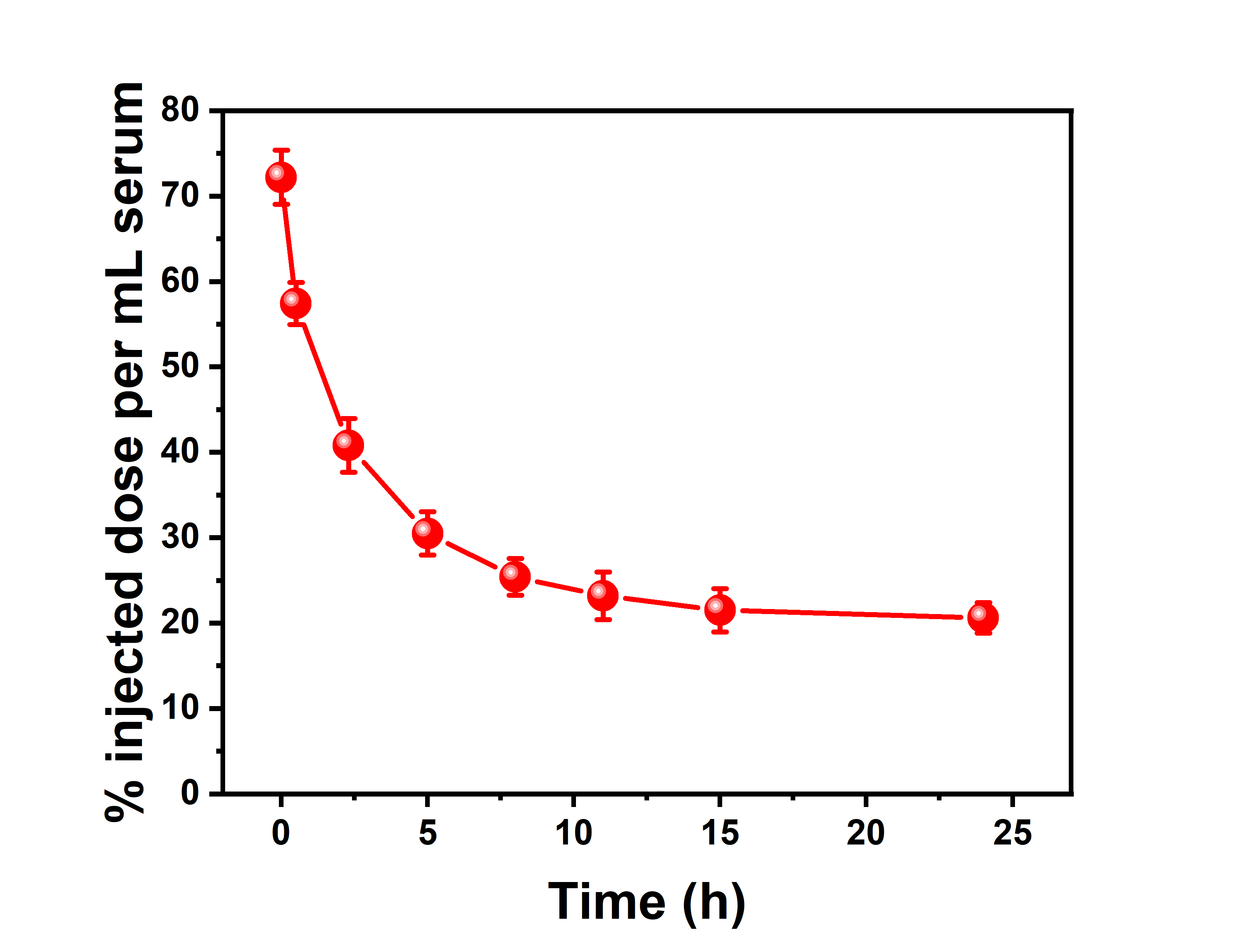


**Figure S3** *In vivo* pharmacokinetics of the USDPFs (5 mg kg^-1^) in mice after intravenous injection.


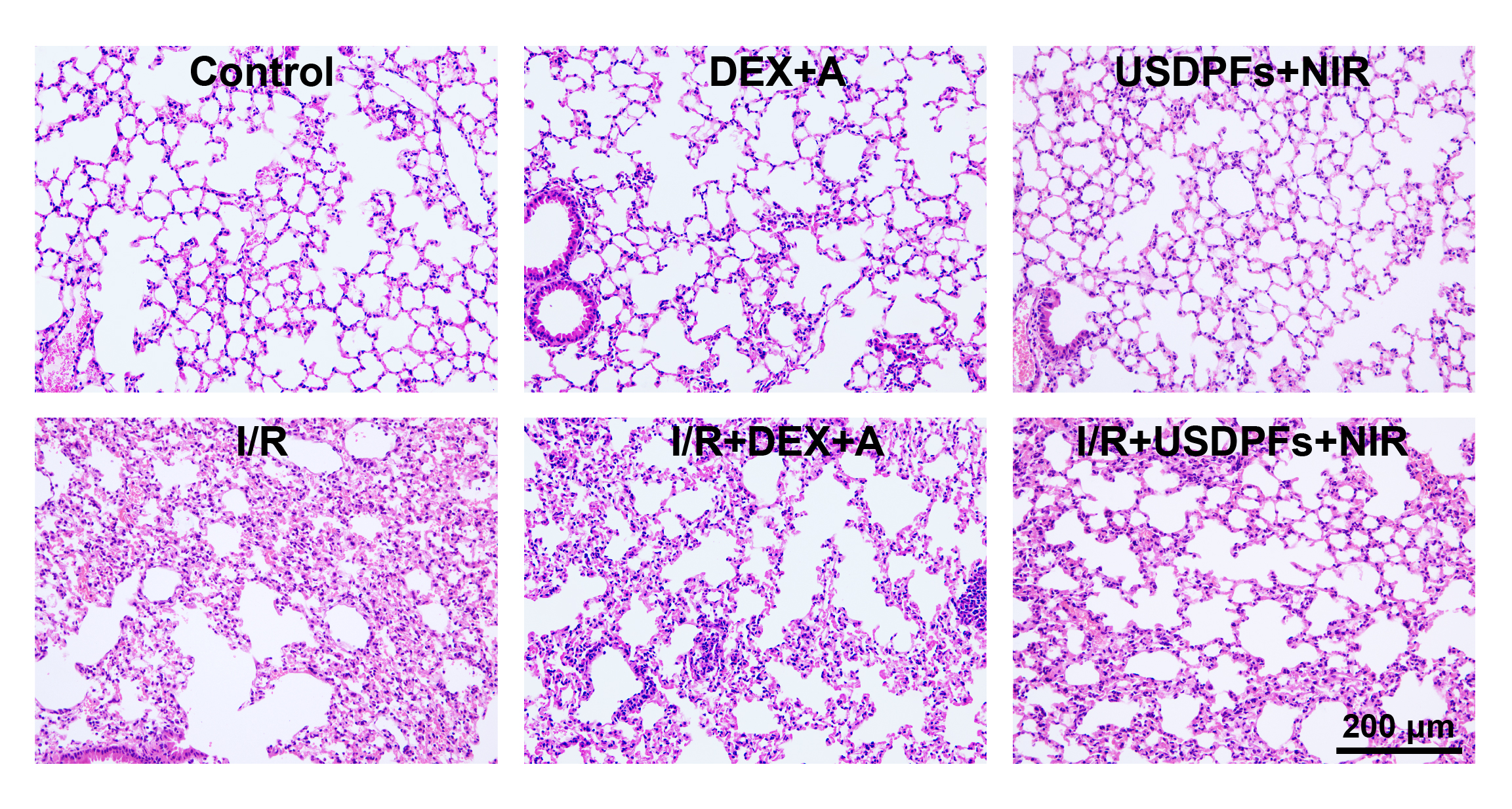


**Figure S3** H&E staining of lung tissue in mice after intratracheal injection of USDPFs (5 mg kg^-1^) and use of dexamethasone aerosol (DEX+A) (× 200, scale bar 200 μm).
